# Identification of ABC transporter G subfamily in white lupin and functional characterization of *L.albABGC29* in phosphorus use

**DOI:** 10.1186/s12864-021-08015-0

**Published:** 2021-10-06

**Authors:** Mehtab Muhammad Aslam, Muhammad Waseem, Qian Zhang, Wang Ke, Jianhua Zhang, Weifeng Xu

**Affiliations:** 1 College of Agriculture, Yangzhou University, Yangzhou, 225009 China; 2 Joint International Research Laboratory of Water and Nutrient in Crops, College of Life Sciences, Fujian Agriculture and Forestry University, Fuzhou, 350002 China; 3 College of Horticulture, South China Agricultural University, Guangzhou, 510642 China; 4 Department of Biology, Hong Kong Baptist University, Stake Key Laboratory of Agrobiotechnology and Chinese University of Hong Kong, Kowloon Tong, Hong Kong

**Keywords:** ABCG subfamily, Phosphorus, White lupin, Rice, Duplication

## Abstract

**Background:**

White lupin (*Lupinus albus*) is a leguminous crop with elite adaptive ability in phosphorus-deficient soil and used as a model plant for studying phosphorus (P) use. However, the genetic basis of its adaptation to low P (LP) remains unclear. ATPase binding cassette (ABC) transports G subfamily play a crucial role in the transportation of biological molecules across the membrane. To date, identification of this subfamily has been analyzed in some plants, but no systematic analysis of these transporters in phosphorus acquisition is available for white lupin.

**Results:**

This study identified 66 ABCG gene family members in the white lupin genome using comprehensive approaches*.* Phylogenetic analysis of white lupin ABCG transporters revealed six subclades based on their counterparts in Arabidopsis, displaying distinct gene structure and motif distribution in each cluster. Influences of the whole genome duplication on the evolution of L.albABCGs were investigated in detail. Segmental duplications appear to be the major driving force for the expansion of ABCGs in white lupin. Analysis of the Ka/Ks ratios indicated that the paralogs of the L.albABCG subfamily members principally underwent purifying selection. However, it was found that *L.albABCG29* was a result of both tandem and segmental duplications. Overexpression of *L.albABCG29* in white lupin hairy root enhanced P accumulation in cluster root under LP and improved plant growth. Histochemical GUS staining indicated that *L.albABCG29* expression increased under LP in white lupin roots. Further, overexpression of *L.albABCG29* in rice significantly improved P use under combined soil drying and LP by improving root growth associated with increased rhizosheath formation.

**Conclusion:**

Through systematic and comprehensive genome-wide bioinformatics analysis, including conserved domain, gene structures, chromosomal distribution, phylogenetic relationships, and gene duplication analysis, the L.albABCG subfamily was identified in white lupin, and *L.albABCG29* characterized in detail. In summary, our results provide deep insight into the characterization of the L.albABCG subfamily and the role of *L.albABCG29* in improving P use.

**Supplementary Information:**

The online version contains supplementary material available at 10.1186/s12864-021-08015-0.

## Background

The ATPase binding cassette (ABC) proteins constitute nucleotide binding domain (NBD) and transmembrane domain (TMD) conserved domains [1] containing superfamily ubiquitous across all living organisms, including plants. ABC transporters gene family encode for a membrane-bounded transporter protein that mediates molecular transportation of soluble proteins across the plasma membrane or among different organelles [2]. With the advent of advanced genomic and bioinformatics techniques, a plethora of ABC transporters has been identified in numerous plant species such as Arabidopsis, soybean, tomato, rice, peppers, pineapple, and Lotus [3–10]. Plant ABC transporter gene family typically distributed to ABCA-ABCI subfamilies, except ABCH subfamily [11]. However, the ABCG subfamily is unique in fungi and plants. Like other ABC subfamilies, the ABCG subfamily is also categorized into full-size ABCG transporters, containing two NBD and two TMD (NBD-TMD2) and half-size transporters containing only one NBD and TMD. The former probably originated by a single duplication of later ABCG transporters group [12].

ABCG is known to be one of the most extensive subfamilies in the plant kingdom and reported to be involved in diverse biological processes such as pathogenicity, cuticle formation [13], transportation of various biological molecules, and hormone transport [14]. ATP-binding cassette (ABC) transporter genes from Arabidopsis, *AtABCG25* and *AtABCD40* exhibited ATP-dependent ABA transport [15]. Mutations in these ABCGs lead to reduced ABA-dependent stomatal closure and severe phenotypes to drought stress [16]. We also reported two white lupin ABCG genes, *L.albABCG36* and *L.albABCG37,* potentially involved in auxin-mediated cluster root (CR) formation under P deficiency [17]. In rice, RCN1/*OsABCG5* involved in ABA-mediated stomatal closure in guard cells [18]. In addition, a half-size *AtABCG22* is involved in the regulation of stomatal responsiveness to change in air humidity in both opening and closing directions [19]. Arabidopsis DSO/ABCG11 transporter affects cutin and suberin Metabolism in reproductive organs and roots tissues [20]. Similarly, *ABCG37* and *ABCG33* functioned as caesium influx carriers in Arabidopsis roots [21]. These suggested the crucial roles of ABCG subfamily members to improve plant growth, development, and nutrients acquisition. However, the identification and characterization of the white lupin L.albABCG transporters subfamily remain to be determined.

White lupin (*Lupinus albus*) is an economically important legume crop belonging to the family *Fabaceae* due to its high nutritious value, high protein and low oil contents [22]. It gained a lot of research attention due to its significant role in improving nutrient mobilization, soil exploration and fertility, and nitrogen-fixing ability [23]. Rhizosheath formation (soil strictly attached with root) is an important root adaptive trait that alleviates nutrient uptake under soil drying (SD) [24]. Phosphorus is an essential macronutrient for plant growth and productivity [25, 26]. It has been reported that rhizosheath with long root hairs having a competitive advantage for P acquisition to relieve P deficiency in barley and wheat [27, 28]. Therefore, a robust rhizosheath can improve P uptake efficiency via root hair growth in water-limited soils. White lupin *L.albABCG37* reported in stimulating CR formation by modulating IBA transport under P deficiency [17], suggesting its role in P acquisition. Rice is an important staple food crop, and 1/3rd of the world’s population relies on it [29]. However, it consumed a massive amount of water and nutrients from the soil, becoming an alarming situation for agriculture sustainability [30]. Several studies reported that rice biomass and yield depend on P availability under acidic soil [31–33]. However, the role of genes involved in improving P uptake efficiency from the soil is scarce. Therefore, an urgency to identify novel candidate genes or plant traits directly involved in improving phosphorus use efficiency would support sustainable agriculture.

This study provided comprehensive information on the white lupin ABCG transporter subfamily and investigated their expression under LP conditions. We analyzed the evolutionary importance of *L. albus* ABCG subfamily with *A. thaliana*, *G. max*, *L. angustifolius,* and *P. vulgaris*. Furthermore, we overexpressed *L.albABCG29* in white lupin and rice to investigate its putative role in improving phosphorus use efficiency under LP hydroponic solution and SD conditions, respectively. Our findings provide cues for future investigation on crop P use improvement.

## Results

### Identification of white lupin ABCG subfamily members

Whole-genome sequence of white lupin was used to identify the ABCG subfamily transporters genes. Arabidopsis ABCG protein sequences were used to search against the white lupin genome to screen out candidate ABCG subfamily members. In total, 66 ABCG transporter genes (Additional file 1) in the whole white lupin genome. The protein length of *L. albus* ABCG genes ranged from 130 (*L.albABCG22*) to 1498 amino acids (*L.albABCG35*). *L. albus* ABCG transporter genes were unevenly distributed across 24 chromosomes except for 18 chromosomes. Of 66 ABCGs, 8 members were located on chromosome 2, accounted for 12.2% of the total LalbABCG transporter gene, followed by 7 on chromosome 20 (10.6%), 6 on chromosome 25 (9.0%), and only 1 gene was located on chromosome 4, 8, 10, 13, 15, 16, 17, and 23 accounted for 1.5%. Moreover, 4 sets of genes were located on chromosome 12, 19, and 24 (6.0%) each, and 3 were found on chromosome 1, 3, 9, 14, and 22 (4.5%), and 2 were found on chromosome 5, 6, 7, 11, and 21 (3.0%) (Additional file 1). Detailed characteristics of the *L. albus* ABCG transporter subfamily, including transcript IDs, gene name, domain type, and in silico subcellular location, is provided in Additional file 2.

### ABCG subfamily is the most diverse in white lupin

**Fig. 1:**
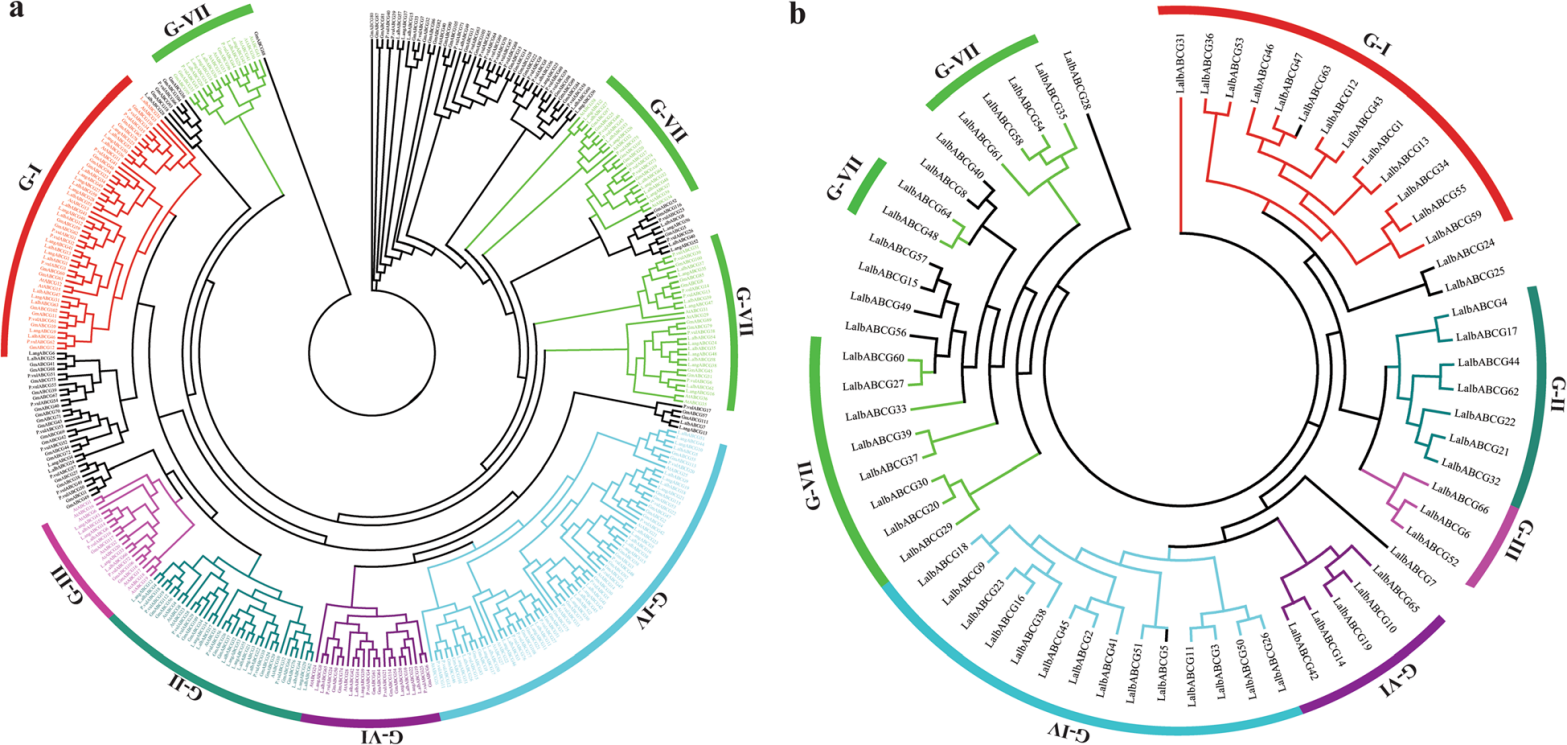
Evolutionary relationship of the ABCG subfamily across different plant species. **a** An unrooted phylogenetic tree of ABCG proteins of *L. albus, L. angustifolius, P. vulgaris, G. max,* and *A. thaliana*. **b** Phylogenetic tree of white lupin ABCG subfamily genes only. The phylogenetic tree was constructed using IQ-TREE software under the LG + I + G model with ML ultrafast bootstrap value (1000). ABCG subfamily clustered into six groups (G-I to G-IV and G-VI) based on *A. thaliana* homologs in each group, represented by a different color arc

Evolutionary analysis of ABCG transporter proteins reveals that the ABCG subfamily is among the most abundant groups among all species (Fig. 1a). ABCG subfamily accounted for 66 members in *L. albus,* 56 *L. angustifolius,* 77 *P. vulgaris,* 117 *G. max,* and 43 *A. thaliana* in the plant lineages. As ABCG subfamily is tremendously diverse in the plants, we are curious to know how this diversity evolved. Phylogenetic analysis of the ABCG subfamily in a broad range of legumes and *A. thaliana* was conducted. The white lupin ABCG transporters proteins clustered into seven groups (I to IV, VI-VII), with other plant homologs. However, group V is absent in white lupin. *A. thaliana*; Group-III shared seven L.albABCG with *AtABCG1*, *AtABCG2*, *AtABCG6*, and *AtABCG16*; Group-IV contained two *A. thaliana* AtABCG (*AtABCG9* and *AtABCG26*) along with 14 ABCG from white lupin. Similarly, Group-VII includes those L.albABCG, which share homology with diverse functional ABCG of *A. thaliana* (Fig. 1a). However, phylogenetic analysis of the white lupin ABCG subfamily (Fig. 1b) follows the above-mentioned phylogenetic clustering (Fig. 1a). Our data suggested that ABCG genes evolved in white lupin to acquire new functions in a very diverse way.

### Gene structure analysis and motif composition of ABCG subfamily

**Fig. 2:**
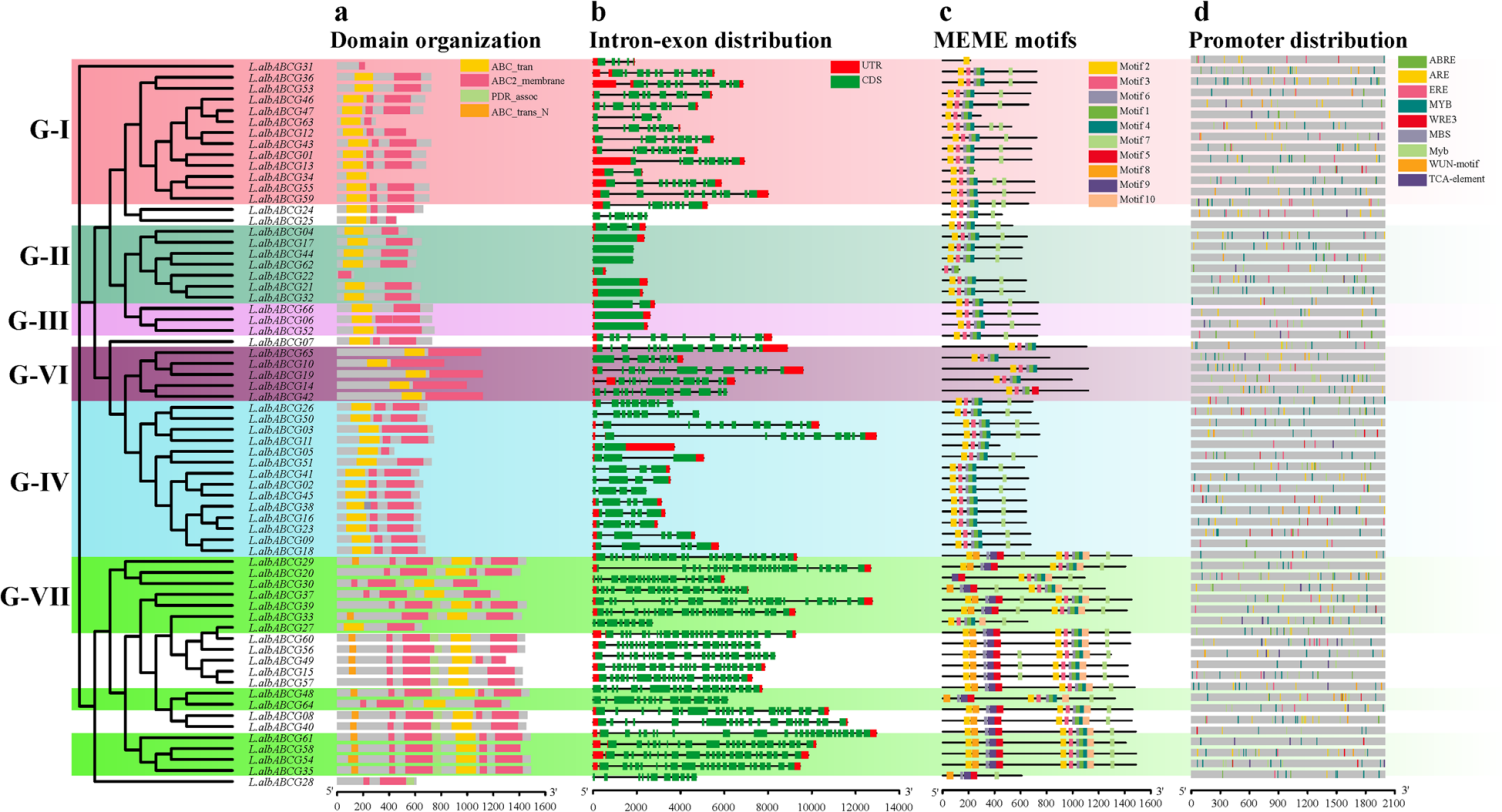
Gene structure and evolutionary analysis of white lupin ABCG transporter subfamily. **a** Conserved domain analysis of ABCG genes. **b** Gene structural analysis of L.albABCG genes, 5′ to 3′ direction represent the orientation of nucleotide sequence and scale at the bottom represent the length of nucleotide (bp). **c** Conserved motif analysis of L.albABCG genes. **d** Predicted cis-regulatory elements of L.albABCG genes. Total 9 randomly distributed promoter cis-regulatory elements identified from 2 kb upstream region of each candidate gene. Each type of *cis*-regulatory element is represented with a different color box at the bottom. The final figure was colored and edited by using Adobe Illustrator CC software

The conserved domain analysis of ABCG transporter proteins revealed four conserved domains exist in two complexes, the first complex as NBD or TMD or contain both (Fig. 2a). These genes have 1–24 exons and scattered over many different chromosomes (Fig. 2b). Intron-exon distribution revealed that closely related ABCG genes were generally displayed a similar gene structure. Eight of 66 ABC transporter genes contained single or 4 exon, including *L.albABCG06, L.albABCG17, L.albABCG21, L.albABCG22, L.albABCG32, L.albABCG44, L.albABCG52,* and *L.albABCG62* or *L.albABCG02, L.albABCG16, L.albABCG23, L.albABCG31, L.albABCG38, L.albABCG41, L.albABCG45,* and *L.albABCG63*, respectively. Moreover, 9 exons are present in *L.albABCG01, L.albABCG13, L.albABCG26, L.albABCG43, L.albABCG47, L.albABCG46,* and *L.albABCG50*, or *L.albABCG20, LalbABCG29, LalbABCG33,* and *LalbABCG39* contained highest number of exons (24) among ABCGs each (Additional file 1 and Fig. 2b). Several closely related genes display similar exons distribution pattern, indicating that these genes belong to the same subclade.

Conserved motifs were analyzed to understand better the global conservation and diversification of white lupin ABCG transporter proteins. A total of 10 conserved motifs were identified and displayed a very diverse distribution pattern validating their phylogenetic classification (Fig. 2c). For instance, ABCG genes in all phylogenetic clades except for G-VI and G-VII contained five and six motifs with some exceptions, respectively. For example, G-I had six motifs with the exceptions of *L.albABCG13, L.albABCG31,* and *L.albABCG63* containing 5, 1, and 4 motifs, respectively. While *L.alABCG62* of G-II contained 3 motifs, and *L.albABCG05* of G-IV contained 5 motifs. All ABCG members in G-VI had 5 motifs, except for *L.albABCG42* of G-VI contained 5 motifs, but motif 4 is exchanged with motif 5. Lastly, G-VII contained all the motifs except for *L.albABCG27* and *L.albABCG28* having 7 and 5 motifs each, respectively. In a few ABCGs some motifs occur twice, including motif 2, 6, and 7 (Fig. 2c). MEME analysis also indicated that 8 of the total 10 conserved motifs overlapping to ABC_trans (NBD) and ABC2_membrane (TMD) domains. Motif-1, motif-3, motif-5, and motif-9 belong to the ABC_trans domain, while motif-2, motif-6, motif-7, and motif-10 belong to the ABC2_membrane domain (Additional file 3).

### *Cis*-regulatory element analysis of *L. albus* ABC transporters

Plant evolves complex signalling mechanisms, including promoter *cis*-regulatory elements related to stress, mediate adaptation to rapidly changing environmental variabilities [34]. The *cis*-regulating elements associated with transcription factors regulate the expression of ABCG transporter genes upon stress conditions. To determine the potential role of *cis*-regulatory elements in *L. albus* ABCGs, 2 kb region upstream promoter of each gene was subjected to *cis-*regulatory elements prediction from the online database PlantCare. The resulting cis-regulatory elements were then compared with available literature. A few key components were selected to draw their distribution.

ABCG transporters subfamily had at least one important *cis*-regulatory element related to stresses. Some important elements involved in stress response or hormone regulation include Abscisic acid-responsive element (ABRE), Auxin responsive element (ARE), Salicylic acid-responsive elements (TCA-element), Wound responsive elements (WRE3), WUN-motif, Ethylene responsive element (ERE), Myb3 binding promoter motif (MBS, Myb), and Auxin responsive element (ARE) were identified in L.albABCG promoter sequences (Fig. 2d). ABRE elements are predicted in 69.70% ABCG genes promoters sequences, while ARE accounted for 86.36%, ERE 63.64%, MBS 56.06%, 69 TCA elements 39.39%, WUN motifs 43.94%, and WRE3 39.39% in all L.albABCGs.

### Synteny and colinearity analysis of *L. albus* ABC transporters genes

**Fig. 3:**
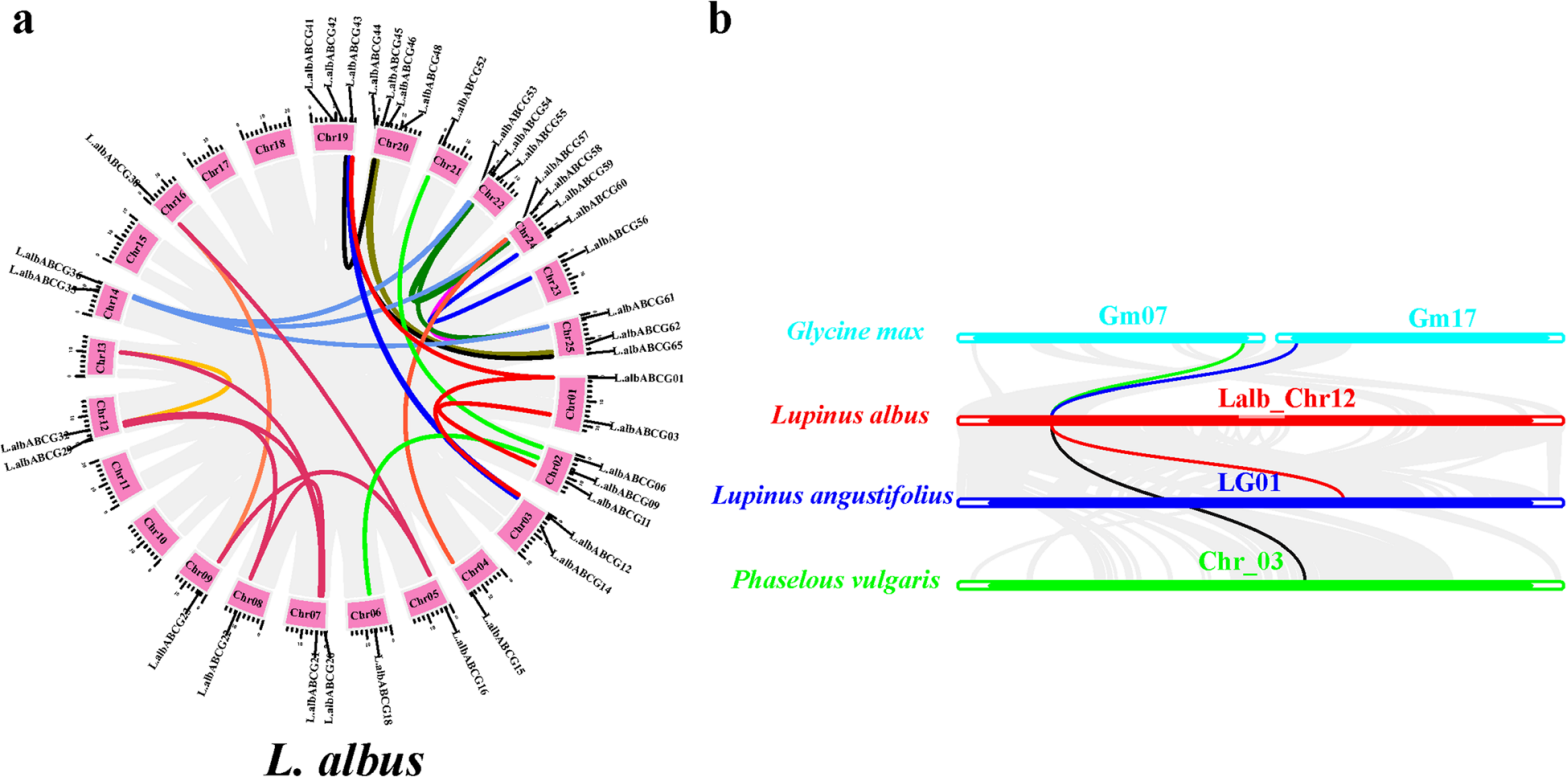
Gene duplication analysis of L.albABCG subfamily. **a** Synteny analysis of the white lupin ABCG subfamily revealed segmental duplication on different chromosomes. Different color lines indicate segmental duplication pairs of L.albABCG between chromosomes. **b** Collinearty map of only *L.albABCG29* between *G. max*, *L. angustifolius,* and *P. vulgaris*. The Blue and green lines indicate *L.albABCG29* syntenic block between *G. max* and *L. albus* chromosome 7/17 and chromosome 12, respectively. The red line indicates the syntenic block of *L. albus L.albABCG29* with the corresponding chromosomes of *L. angustifolius* (chromosome 1, LG01) and blackline *P. vulgaris* (Chromosome 3, Chr_03)

Gene duplication plays a pivotal role in gene family expansion [35]. In the current study, a total of 31 paralogues gene pairs in the L.albABCG subfamily were identified (Fig. 3a). Out of 31 L.albABCG transporter gene pairs, 29 L.albABCGs were segmental duplication pairs (involving 38 L.albABCG genes), and 2 L.albABCG were tandemly duplicated pairs (involving 4 L.albABCG genes) in the whole *L. albus* genome (Additional file 4). These segmental duplication pairs were randomly distributed on all chromosomes, and the maximum number of genes were located on chromosomes 20 and 24. Contrastingly, chromosomes 3 and 20 contained single pairs of tandemly duplicated L.albABCG genes (Additional file 1). These results suggest that segmental duplication was the major driving force for ABCG gene expansion in the *L. albus* genome.

Moreover, we constructed comparative synteny maps of *L. albus* with *A. thaliana, L. angustifolius, P. vulgaris,* and *G. max.* A total of 25 orthologous pairs of segmentally duplicated L.albABCG were identified by comparing with *A. thaliana* genome (Additional file 5a & 6), distributed on 13 white lupin chromosomes. The maximum number of L.albABCG genes of orthologous pairs were located on chromosomes 2 and 25. Moreover, 31 and 44 orthologous pairs of segmental duplications were detected in *G. max* (Additional file 5b & 7) and *L. angustifolius* genome (Additional file 5c & 8), respectively, whereas no tandem duplication was found. Additionally, *L.albABCG29* was paired with two *L. angustifolius* genes (XP_019455889.1, XP_019463336.1) (Additional file 9), while no duplication pair was found with *A. thaliana* and *G. max* may imply that *L.albABCG29* may exist before ancestral divergence. Furthermore, the Ka and Ks substitution rate was calculated to determine the selection pressure of gene pairs. We found that all segmental and tandem duplication pairs of different species had a Ka/Ks ratio < 1, implying that *L. albus* ABCG transporters subfamily genes may experience purifying selection during evolution (Additional files 4 & 6, 7 and 8).

To further infer the effect of evolutionary pressure on *L. albus* ABCGs, we constructed comparative colinearity maps of *L. albus* among five different representative plant species, including *L. angustifolius, P. vulgaris, G. max,* and *A. thaliana* (Additional file 9). A total of 130 orthologous pairs exhibited a colinear relationship between *L. albus* and *G. max,* followed by 96 with *L. angustifolius*, 71 with *P. vulgaris*, and 34 with *A. thaliana*. Moreover, to further infer the evolutionary significance of *L.albABCG29,* a collinearity map between *L. albus L.albABCG29* with *G. max, L. angustifolius,* and *P. vulgaris* was generated. *L.albABCG29* form two collinear pairs with *G. max* (Glyma07g36160, Glyma17g04350), and single pair with *L. angustifolius* (XM_019600344.1)*,* and *P. vulgaris* (XM_007154390.1). Whereas no collinear block was detected with *A. thaliana* (Fig. 3b)*.* The functional characterization of *L.albABCG29* can provide a more valuable gene functional reference for legumes growing under phosphorus deficient conditions.

### *L.albABCG* genes are differentially expressed under low phosphorus

**Fig. 4:**
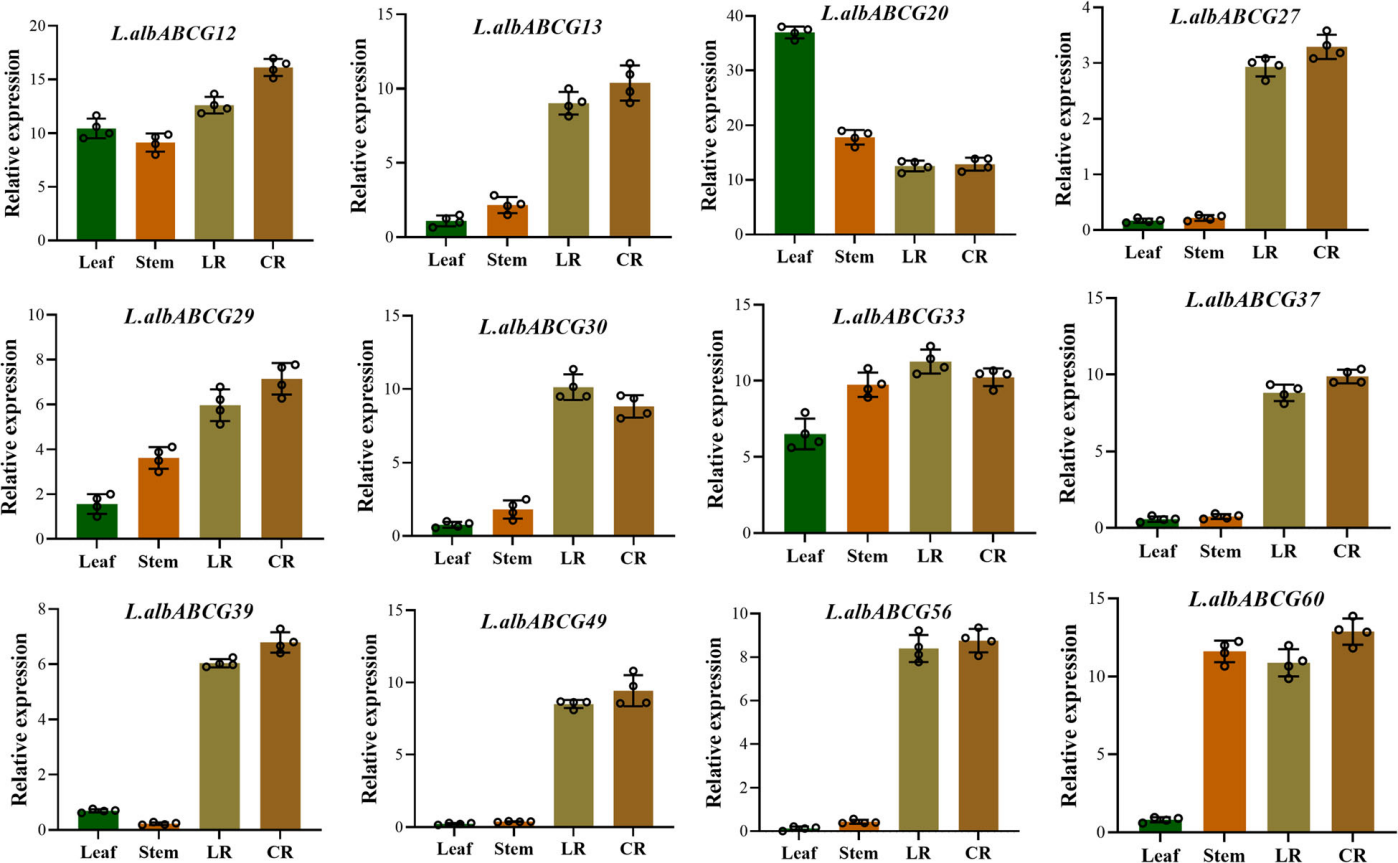
Tissue-specific expression pattern of selected L.albABCG genes under low P. in LR; lateral root, CR; cluster root was assessed. Four independent replicates were used to calculate expression in each tissue

To elucidate the biological significance of white lupin ABCG in phosphorus transport, we examined the gene expression pattern of ABCG subfamily members among different *L. albus* tissues grown under CK and LP conditions using our previously generated RNA-seq data. The expression pattern of L.albABCG in different tissues revealed that the majority of genes were sensitive to LP (Additional file 10). For instance, *L.albABCG46, L.albABCG47,* and *L.albABCG48* showed higher expression in leaf (L) under CK compared to LP conditions. Most of the genes had a relatively lower expression in the shoot (S), while few genes were expressed under both CK and LP conditions. In CR, *L.albABCG29* was highly expressed under LP conditions, while *L.albABCG17, L.albABCG52, L.albABCG58,* and *L.albABCG66* had moderate expression levels. Four genes *L.albABCG3, L.albABCG20, L.albABCG26,* and *L.albABCG35* were highly expressed in L and *L.albABCG38,* and *L.albABCG64* has only a highly expressed root tip (RT) under LP condition. Contrastingly*, L.albABCG2, L.albABCG5, L.albABCG7, L.albABCG9, L.albABCG14, L.albABCG15, L.albABCG16*, *L.albABCG18, L.albABCG25, L.albABCG37, L.albABCG43, L.albABCG44, L.albABCG60,* and *L.albABCG65* in S under CK and LP stresses, respectively (Additional file 11). To further validate the expression of L.albABCG, L.albABCGfour duplication pairs and others from phylogenetic clade G-VII were selected to investigate their expression in different white lupin tissues under low P. The results showed that overall expression trends of these genes by RT-qPCR were consistent with that of RNA-seq analysis. All the L.albABCG genes showed the highest expression levels in the lateral root (LR) and CR except *L.albABCG20,* which had the highest expression in the leaf (Fig. 4). RNA-seq data and RT-qPCR analysis showed that most genes showed higher expression in CR and lower in LR under LP, indicating ABCG transporters may be involved in mitigating P related stresses.

### Overexpression of *L.albABCG29* improves phosphorus uptake in white lupin

**Fig. 5:**
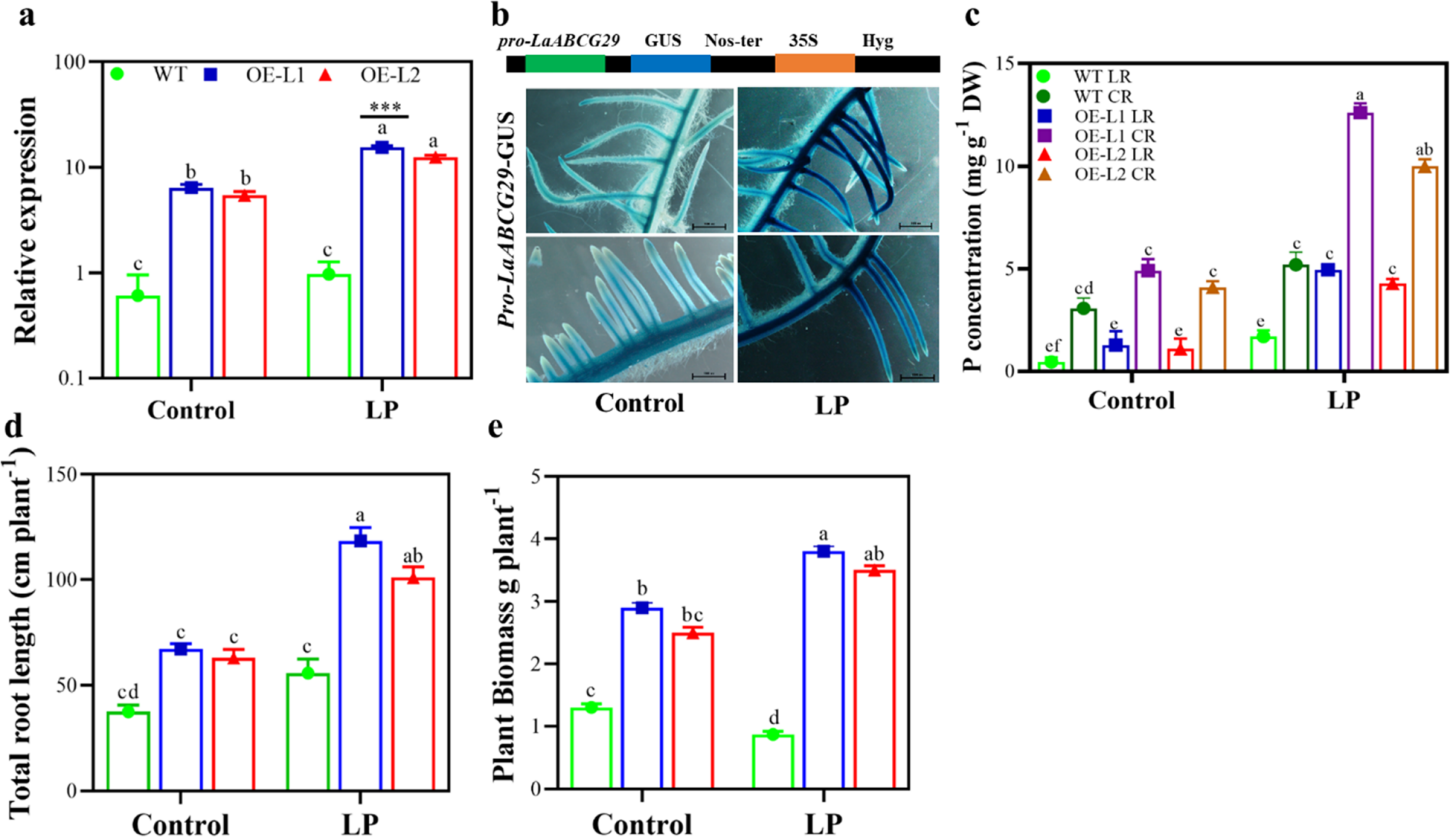
Phenotypic characterization of *L.albABCG29* in white lupin grown under phosphorus sufficient (CK) and low phosphorus (LP) conditions. **a** Relative expression of *L.albABCG29* in transgenic white lupin hairy roots **b** schematic representation of the GUS vectors for transient expression (top panel). The native promoter of *L.albABCG29* drives GUS expression in transgenic *L. albus* roots under CK and LP conditions (lower panel), **c** phosphorus concentration (mg P g^− 1^, DW) in different parts of white lupin root, **d** total root length (cm), **e** total plant biomass (g plant^− 1^) of transgenic (OE-L1/L2) and wild type (WT) white lupin. Different letters show significant differences of mean ± SD of four replicates at *P* < 0.01 significance level. OE-L1, Overexpression Line 1; OE-L2, Overexpression Line 2

Previously, we reported that *L.albABCG29* might act as a critical gene in P acquisition under low P. Here, we also found continuous increasing expression of *L.albABCG29* in different tissues and peaked expression in CR. We generated transgenic hairy roots overexpressing *L.albABCG29* in white lupin. We found significantly increased expression under LP conditions compared to the non-transgenic roots (Fig. 5a). To precisely analyze the expression of *L.albABCG29* in the roots under P variability, transgenic lines resulting in the expression of *L.albABCG29* promoter::GUS was generated. Staining of transgenic plant root revealed elevated expression of *L.albABCG29* under low P (Fig. 5b). After 6-weeks of transformation, transgenic CR and LR of *L. albus* were collected to measure phosphorus content. We found that CR had a higher phosphorus concentration followed by LR under LP conditions (Fig. 5c). LP condition showed enlarged root growth in both transgenic lines (Fig. 5d) and improved overall plant biomass (Fig. 5e), indicating that transgenic roots have a role in nutrient translocation from root (transgenic) to shoot (non-transgenic), ultimately improves plant growth.

### *L.albABCG29* encourage phosphorus uptake in rice

**Fig. 6:**
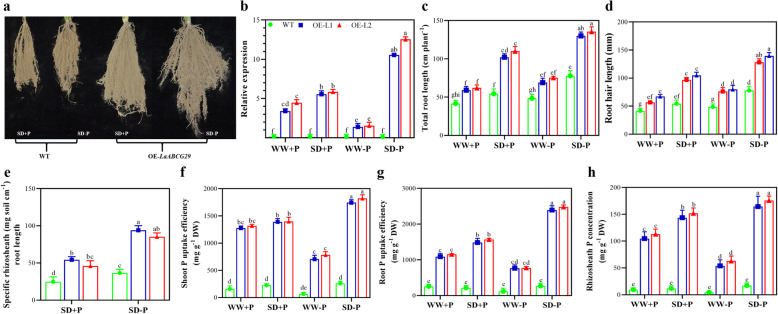
Phenotype of *L.albABCG29* overexpression in rice under combined water and phosphorus-deficient soil conditions. **a** Phenotypes of rice plant rizhosheath under SD + P and SD-P in both WT and *L.albABCG29* overexpression lines, **b** relative expression level, **c** total root length (cm), **d** root hair length (mm), **e** specific rhizosheath weight (mg cm^− 1^ root length), **f** phosphorus uptake efficiency in the shoot (mg P plant^− 1^), **g** phosphorus uptake efficiency in the root (mg P plant^− 1^), **h** rhizosheath P concentration (mg P g^− 1^, DW). Different letters show significant differences of mean ± SD of four replicates at *P* < 0.01 significance level. SD + P; soil drying with phosphorus, SD-P; soil drying without phosphorus, WW + P; well-watered with phosphorus, WW-P; well-watered without phosphorus; OE-L1, Overexpression Line 1; OE-L2, Overexpression Line 2

Due to the lack of *L. albus* stable transformation protocol, we then overexpressed *L.albABCG29* in rice. *L.albABCG29* overexpression transgenic rice plants were used to grow under four different (WW + P, SD + P, WW–P, and SD–P) soil treatments. Plants were harvested after 45 days of growth, and rhizosheath formation was observed only under SD treatments (SD + P and SD-P) (Fig. 6a), no rhizosheath was developed under WW conditions. We found that SD–P showed higher expression of *L.albABCG29* in transgenic rice lines than WT among all four soil treatments (Fig. 6b). SD–P treatment showed increased rhizosheath formation in transgenic lines compared to SD + P (Fig. 6e), suggesting that OE*-L.albABCG29* involved enhanced rhizosheath formation via extended root structure and hair growth (Fig. 6c-d). Additionally, we observed that OE-*L.albABCG29* showed a higher P concentration compared to WT among all tissues. Root showed higher P uptake under SD–P (Fig. 6g). Shoot P uptake was also higher under SD–P in the overexpression line (Fig. 6f), indicating the phosphorus uptake improved under combined SD and LP conditions. Significant differences were noted among rhizosheath P concentration of OE-*L.albABCG29* and WT plants under four different soil treatments (Fig. 6h). The data suggested that OE-*L.albABCG29* is associated with increased P uptake in *O. sativa* by improved rhizosheath formation and root growth, especially under SD–P soil treatment.

## Discussion

ABC transporters are ubiquitous membrane-bound proteins [2, 36] present in all prokaryotes, from plants to animals [3, 37]. Comprehensive genome-wide investigation of the ABC transporter gene family among several plant species provides deep insight in identifying their regulatory mechanism and functional characterization to stress responses [38]. Phylogenetically ABC gene family is divided into distinct subfamilies, including the ABCA-ABCI subfamilies, except the ABCH subfamily [8, 9, 11]. Among these subfamilies, the ABCG subfamily is tremendously diverse and encode either 577–1107 amino acids (aa) for half-size or 1382–1469 aa for full-size ABCG transporters proteins [12]. ABCG transporter proteins are functionally diverse and ubiquitous in all organisms, including animals and plants. Several studies have been reported that ABCG was the largest subfamily of the ABC gene family in plants but needed to be functionally characterized [2]. These genes have 1–24 exons and distributed on different chromosomes. In this study, we identified ABCG subfamily members in white lupin (Additional file 2). Phylogeny of the ABCG subfamily in plant lineage reveals several exciting insights [39]. Our phylogenetic study validated those findings; for instance, white lupin ABCG transporter proteins were clustered with *A. thaliana* ABCG proteins. Unlike *A. thaliana,* L.albABCG proteins were diversified into six clusters (Fig. 1a). We speculated that proteins sharing a similar ancestral origin might have similar functions. This suggested that L.albABCG clustered with *A. thaliana* homologs may share some functional similarities which need to validate.

Duplication of the gene on the individual chromosome, between different chromosomes, or even entire genomes may be a major driving force in building up gene diversity during genome evolution [40]. Our findings showed that segmental duplication events of the ABCG subfamily were more frequent within the *L. albus* genome (Additional file 4 & Fig. 3a) comparing with *G. max*, *L. angustifolius*, and *A. thaliana* (Additional file 5). For instance, we found a total of 66 ABCG members in *L. albus* (Additional file 4), while 43 in *A. thaliana* (Additional file 6) were previously reported [9, 41], indicating expansion of ABCG subfamily in legumes. Additionally, we compared gene duplication events of ABCG subfamily genes within the *L. albus* genome and with *A. thaliana, G. max,* and *L. angustifolius* genomes (Additional file 5). A total of 29 segmental duplication pairs (involved 38 L.albABCG genes) and 2 tandem duplication pairs (involved 4 L.albABCG genes) were identified within the *L. albus* genome (Additional file 4). A total of 31 segmental duplication pairs were found between *L. albus* and *G. max* genome (Additional file 7), while 44 segmental pairs by comparing with *L. albus* with *L. angustifolius* genome (Additional file 8). Contrastingly, 25 segmental duplication pairs were shared by *L. albus* and *A. thaliana* genome (Additional file 6). More specifically, the *L.albABCG29* pair was found in both tandem and segmental duplication, indicating that duplication events could be a major cause of ABCG subfamily expansion. Most of the members of ABCG transporter subfamily originated by segmental gene duplications, which suggests that segmental duplication may contribute as a major driving force for ABCG transporters evolution in the *L. albus* genome.

ABCG subfamily members are involved in regulating many biological processes. For instance, ABCG transporters are known to be involved in the metal detoxification process. Overexpression of *ABCB25* showed enhanced resistance to polluted cadmium conditions in *A. thaliana* [42]. *AtABCG37* is a close homolog of the *L.albABCG29*, showed IBA and 2,4-D emission into the soil due to its locality on the plasma membrane [43]. Pighin et al. [44] reported that ABCG subfamily involves the transportation of lipids. Gene function prediction and regulation are mainly determined by identifying *cis-*regulatory elements in the promoter region [45]. Several transcription factors and RNA polymerase II enzyme binds with the TATA box that forms a transcription initiation complex to regulate the transcription process [46]. TCA element involved in salicylic acid responsiveness and WUN motif related to wound responsiveness. Several other stress-related elements were identified, involving the DRE element involved in dehydration responses. Additionally, *cis*-regulatory elements related to hormonal responses, such as ABRE and ARE responsive elements, were identified (Fig. 2d). Our finding collectively showed the diverse functionality of the ABCG subfamily among different cellular, hormone signalling mechanisms to improve plant growth, development, and stress response.

The release of the high-quality *L. albus* genome makes it readily available for researchers to discover important candidate genes related to specific agronomic traits [17, 47]. Transcript expression of most of the root regulatory network related genes was significantly increased under LP conditions, particularly in CR tissues [48], suggesting that LP activates several genes involved in alleviating nutrient and water stress. Similarly, we performed RNA-seq analysis [17] of different *L. albus* tissues grown under LP revealed a total of 2128 DEGs (differentially expressed genes). A total of 904 genes were found in P-deficient CR compared to P-sufficient R to understand plant acclimation responses. For example, members of the bHLH transcription factor family (14 genes) showed increased expression in P-deficient CR [49]. We also found that *L.albABCG37* (rename as *L.albABCG29* in the present study) was also upregulated under LP in white lupin root [17]. Our RNA-seq data showed that L.albABCG subfamily genes have higher expressions in root tissues than shoot under LP conditions (Additional file 12), which implies that these ABCGs play a significant role in plant adaptation to limited nutrient/P stress. These findings indicate that plants require ABCG proteins for maintaining root growth and nutrient uptake related processes. Notably, *L.albABCG29* had higher CR expression under LP conditions than other plant tissues, which was further validated by overexpression in rice. Overexpression of *L.albABCG29* in white lupin hairy root improved plant biomass under LP conditions (Fig. 5e), while in *O. sativa* its overexpression displayed increase rhizosheath formation via extending root length and root hair growth (Fig. 6c-d). Enhanced rhizosheath formation is directly associated with improved PUE under SD-P soil compared to WW-P (Fig. 6f-h). Our study indicates that *L.albABCG29* promote root growth, increase rhizosheath formation, which is associated with improved phosphorus acquisition from the soil, and ultimately improved overall plant biomass. *L. albus* ABCG subfamily analysis provides comprehensive information on gene function, which may serve as a base for their role in generating P efficient crop that may emerge as a promising approach to meet plant nutritional demand under LP conditions.

## Conclusion

ABC transporter gene families are more diverse and ubiquitous gene families among all living organisms. We identified *L. albus* ABCG transporter genes classified into six clusters. Variation in genes structural features supports evolutionary relationships based on their domain homology and phylogeny. Furthermore, gene duplication played a vital role in the expansion of L.albABCG subfamily in *L. albus*. RT-qPCR validation revealed that *L.albABCG29* showed higher expression in CR under low P conditions, indicating its role in plant nutrients acclimation responses to starvation. Overexpression of *L.albABCG29* improves P uptake in transgenic rice by improving root growth associated with increased rhizosheath formation. However, our data suggest that ABCGs show function redundancy when exposed to specific environmental stress. Collectively, this study provides a comprehensive role of *L.albABCG29* in improving P uptake in plants. Functional characterization of essential ABCG subfamily transporters genes in response to nutrient and water stress will give helpful information for the generation of nutrient efficient and stress-resistant crop production.

## Materials and methods

### Identification of L.albABCG subfamily

To identify the ABCG gene subfamily in *L. albus*, the reported ABCG peptide sequences of *A. thaliana* [50] retrieved from the Phytozome (V12) database [51] were used as query sequences to perform BLASTP searches against white lupin genome (https://www.whitelupin.fr/) [52–54]. The deduced protein sequences were validated in HMMER [55] for the presence of ABC transporter domain (PF00005) [56], manually verified in Simple Modular Architecture Research Tool (SMART) [57] and NCBI Conserved Domain Tool (Batch CD-Search Tool) [58]. Finally, all the non-redundant sequences and those lacking conserved domain or motif were removed, and the remaining sequences were used for further analysis. For each *L. albus* ABCG protein, in silico subcellular localization was predicted using WoLF PSORT [59].

ABC transporter protein sequences of *G. max,* [8, 50], *L. angustifolius,* and *P. vulgaris* were retrieved from Phytozome. *L. albus* protein sequences and *A. thaliana*, *G. max, L. angustifolius,* and *P. vulgaris* were aligned using a muscle program [60]. The resultant multiple sequence alignment was used to construct an unrooted maximum-likelihood using IQ-TREE [61] and bootstrap adjusted to 1000 replicates. Finally, the tree was visualized and annotated in the MEGA program [62].

### Gene structure and *cis*-regulatory element analysis

For ABCG genes intron-exon distribution, the genomic and corresponding nucleotide coding sequences were downloaded from the *L. albus* genome database [54] and submitted to the Gene Structure Display Server (GSDS) [63]. Conserved motifs were predicted using MEME server [64] with the following parameters; a maximum number of motifs was 10, the optimum width was adjusted between 6 to 100 bp (base pairs). To predict *cis*-regulatory elements in L.albABCG genes promoter sequences, 2 kb upstream nucleotide sequences from the transcription activation site (ATG) were downloaded and submitted to PlantCARE [65].

### Chromosomal distribution and gene duplication analysis

The white lupin genome is mapped across 25 chromosomes [17]. The chromosomal distribution of L.albABCG subfamily genes was visualized using MapGene2chromosome (http://mg2c.iask.in/mg2cv2.1/). To examine gene duplication events, the ABCG transporters gene in white lupin and other plants, including *A. thaliana, G. max,* and *L. angustifolius*, MCScanX [66] was used. The gene duplication pairs were visualized in Circos [67]. The whole-genome sequences of different plant species, including *L. albus, L. angustifolius, P. vulgaris, G. max,* and *A. thaliana,* were used to analyze the collinear relationship. The detected syntenic blocks were visualized using Dual Synteny Plotter [67]. Furthermore, Ka and Ks substitution rates were calculated each syntenic pair using Ka/Ks calculator [68].

### Expression analysis of ABCG subfamily in white lupin

White lupin seeds were grown under phosphorus-sufficient (CK, 0.25 mM KH_2_PO_4_) and low phosphorus (LP, 0 mM KH_2_PO_4_) hydroponic solution (1.75 mM K_2_SO_4_, 1.25 mM MgSO_4_, 0.5 mM Ca(NO_3_)_2_, 20 μM Fe(III)-EDTA, 25 μM H_3_BO_3_, 1.5 μM MnSO_4_, 1.5 μM ZnSO_4_, 0.5 μM CuSO_4_, 0.025 μM (NH_4_)_6_Mo_7_O_24_, and 0.25 mM KH_2_PO_4_). To determine the expression profile of ABCGs in *L. albus* tissues, different plant parts such as leaf (L), stem (S), root (R), root tip (RT), and cluster root (CR) were collected from 45 days hydroponically grown *L. albus* plants. The harvested tissues/parts were stored at − 80 °C for further analysis. The expressions of the L.albABCG subfamily in *L. albus* were analyzed from previously obtained RNA-seq data deposited to NCBI GEO (Gene expression omnibus) number GSE31132 [69]. Genes expression was visualized in heatmap generated with R (version 3.8) using the RColorBrewer package.

### Hairy root transformation of white lupin

The full-length coding sequence of *L. albus L.albABCG29* (Lalb_Chr12g0200641, 4,362 bp) was amplified with forwarding primer 5`-BamHI-CGGGATCCATGGCTCAGCTGGTTGGT-3` and reverse primer 5`-SmaI-CCCCCGGGTTATCTTTTCTGGAAGTTGAGGTTT-3`. The amplicon was digested with BamHI and SmaI restriction enzymes and cloned into corresponding multiple cloning sites in modified pFGC5941 vector harboring 35S promoter and kanamycin and *bar* (glufosinate) gene for selection. Additionally, a 2 kb native promoter sequence of *L.albABCG29* was amplified using forward 5`-PstI-AACTGCAGGAATGAGATGAAGAGCCTTCC-3` and reverse 5`-BamHI-CGGGATCCCTC AACACAATACTAAGGACC-3` primers. The amplified promoter region was then subsequently cloned into the pCAMBIA1301 vector by replacing its 35S promoter harboring the GUS gene and hygromycin as a stable marker. Both constructs were then transformed into *Agrobacterium rhizogenic* strain (K599) (Shanghai Weidi Biotechnology, China) using the freeze and thaw method as described by Liu et al. [70].

The surface-sterilized seeds of *L. albus* (cv. Amiga) were grown on ½ MS medium (pH 5.6) supplemented with 1% sucrose. After 3-days of germination, the elongated root was carefully excised with sterilized scalpels and sharp blades. The injured point was immediately dipped into *Agrobacterium* suspension solution for 30 mins. The infected seedling was then shifted to co-culture media (½ MS media augmented with 1% sucrose (w/v), 150 μmol AS, 0.8% agar, at pH 5.5) and kept in the dark chamber for 3-days. Explants were washed with sterilized water mixed with Carbenicillin 400 mg ml^− 1^ and dried on sterilized filter paper inside the laminar flow chamber. The transformed seedlings were shifted to ½ MS solid media and 25 mg ml^− 1^ kanamycin in capped glass jars and kept in a sterilized growth chamber. After 14 days, hairy roots were beginning to develop, and transgenic roots were confirmed by performing qPCR using *bar* gene forward 5`-ATATCCGAGCGCCTCGTG-3` and reverse 5`-CACGCAACGCCTACGACT-3` primers. After 20 days of transformation, hairy roots were transferred to CK (0.25 mM KH_2_PO_4_) or LP (0 mM KH_2_PO_4_) hydroponic solution. Finally, CRs and LRs were sampled after 4-weeks of treatment to proceed with further analysis.

### GUS staining and transient expression

Transgenic white lupin hairy roots grown under CK and LP nutrient solutions were collected and dipped into GUS staining buffer augmented with 5-Bromo-4-chloro-3-indolyl β-D-glucuronide (X-Gluc) substrate (Jefferson, 1989) and kept at 37 °C for overnight. GUS staining was observed under Differential interference contrast (DIC) microscope, and the images were captured with a DXM1200 digital camera (Nikon).

### Rice transformation and water/phosphorus treatments

The full-length cDNA sequence (4362 bp) of *L.albABCG29* was amplified (using the same primers mentioned in section 2.5) and then cloned into pBWA(V)HS overexpression vector harboring 35S promoter and hygromycin gene as a selection marker. Finally, pBWA(V)HS::*L.albABCG29* transformed into *A. tumefaciens* (EHA105, BioRun, Wuhan, China). Transgenic plants were screened by performing RT-qPCR analysis. For phenotypic characterization of *L.albABCG29* transgenic homozygous lines (T3 lines) were generated in rice plants. The soil used to conduct this experiment was acquired from paddy rice field of Huayang, Jiangxi Province, China (115°09′32′′E, 28°32′29′′N). The physicochemical properties of collected soil were as follows: total K, 27.7 g kg^− 1^; total N, 1.75 g kg^− 1^; total P, 0.65 g kg^− 1^; organic C, 20.5 g kg^− 1^; exchangeable K, 92.0 mg kg^− 1^; and Olsen P, 42.6 mg kg^− 1^. The plant pots (designated as +P, with phosphorus) were supplemented with nutrient solution containing 1.75 mM K_2_SO_4_, 1.25 mM MgSO_4_, 0.5 mM Ca(NO_3_)_2_, 20 μM Fe(III)-EDTA, 25 μM H_3_BO_3_, 1.5 μM MnSO_4_, 1.5 μM ZnSO_4_, 0.5 μM CuSO_4_, 0.025 μM (NH_4_)_6_Mo_7_O_24_, and 0.25 mM KH_2_PO_4_. The -P (without P) soil was supplied with the same nutrient solution as stated above, but only with 0 mM KH_2_PO_4_ throughout the growth period. WW treatment represents well-watered pot (top 5 cm layer on pot), and SD represents the soil moisture (20%) throughout the study. Soil treatments were designed as WW + P (well-watered and with P), WW–P (well-watered and without P), SD + P (soil drying with P), and SD-P (soil drying without P). The soil was completely dried and stained through a 4 mm mesh to ensure the soil moisture percentage and homogeneity. A total of 1.8 kg of soil was added to each pot, WW treatments were performed every day to maintain a water level up to 5 cm of the pot, and in SD treatments, 100 ml water was added every third day. Water moisture and phosphorus status were maintained under all treatments throughout the experiment.

### cDNA synthesis and quantitative real-time polymerase chain reaction

To investigate the expression variation of *L.albABCG29* under phosphorus variability supplemented in liquid culture and soil, *L. albus* and *O. sativa* root samples were collected, respectively. The total RNA of selected plant tissues was extracted using Trizol reagent (Invitrogen) as described by Yockteng et al. [71]. RNA integrity and quantity were confirmed by running on 2% gel electrophoresis and Nanodrop (ND-1000) spectrophotometer, respectively. The first strand of cDNA was synthesized from purified RNA samples using PrimeScriptTM RT Reagent Kit (TaKaRa, DALIAN) following the manufacturer’s instructions. Gene-specific primers for *L.albABCG29* and other selected ABCG genes were designed in Prime Quest online database https://www.idtdna.com/PrimerQuest/Home/Index (Additional file 12). The RT-qPCR reaction was performed in a 20 μL reaction volume containing 1 μL of cDNA, 1 μL of each primer, 10 μL of SYBR Green mix (TAKARA), and ddH_2_O up to 20 μL under the following program: 95 °C for 3 min; 32 cycles at 95 °C for 15 s, 60 °C for 15 s and 72 °C for 30 s; 72 °C for 10 min (Bio-Rad, CFX Connect Real-Time PCR Detection System).

### Plant biomass measurement

To determine the effect of each treatment on plant biomass in overexpression transgenic (OE-*L.albABCG29*) and wild type rice plants (Zhonghua 11, Zh-11) shoot and root samples (after 45 days of growth) were harvested and kept at 60 °C incubator for 3 days to dry completely. The dried plant tissues were weighed to calculate the total plant dry weight.

### Rhizosheath collection and phosphorous measurements

Rice plants were carefully disassembled from pots as described in Aslam et al. [72]. The plant roots were systematically shaken so that only soil adhering to roots was collected and designated as rhizosheath soil. Roots attached with rhizosheath soil were washed in a tray, and the soil was dried in an oven at 60 °C for 3–4 days. After drying, soil weight was calculated and designated as total dry rhizosheath weight. To carefully determine the phosphorus concentration in different plant tissues such as roots, stem, and leaf. All the collected samples were washed with ddH_2_O and dried at 60 °C incubator for 3 days. The dried samples were then ground into a fine powder and digested in H_2_O_2_ and H_2_SO_4_. Phosphorous concentration in digested tissues was measured spectrophotometrically at 700 nm using an ammonium molybdate method [73]. PUE (phosphorus uptake efficiency) was determined using this formula presented by Irfan et al. [74].

$$ \mathrm{Phosphorus}\ \mathrm{uptake}\ \mathrm{efficiency}\ \left(\mathrm{PUE}\right)=\mathrm{P}\ \mathrm{concentration}\ \left(\mathrm{mg}\right)\times \mathrm{Plant}\ \mathrm{dry}\ \mathrm{weight}\ \left(\mathrm{mg}\right) $$

## Supplementary Information

**Additional file 1:.** Chromosomal distribution of White lupin ABCG transporter subfamily. Names of chromosomes are mentioned on left side, genes linked with purple line indicates tandem duplication, and the chromosome size scale is in Mb. Different colours inside the chromosome bar represent genes density of each chromosome.

**Additional file 2: **Detailed information of *L. albus* ABCG subfamily genes

**Additional file 3:.** Conserved protein sequences of ten motifs identifed in ABCG subfamily of white lupin. Motif scan and sequence logos were generated in linux based MEME program.

**Additional file 4: **Whole genome duplication of ABCG subfamily and Ka/Ks ratios in the *L. albus* genome

**Additional file 5: **Duplication gene pairs identified with Synteny analysis in *L. albus* ATPase-binding cassette transporters (ABC) gene family. **a** duplicated gene pairs in *L. albus* vs *A. thaliana* **b** duplicated gene pairs in *L. albus* vs *G. max*. **c** duplicated gene pairs in *L. albus* vs *L. angustifolius*. Different color lines exhibited paralogous pairs of ABC transporter genes and their subsequent location on chromosomes. The shaded gray background shows the synteny pairs of the whole-genome and respective chromosome numbers are labeled outside the circle. Pink blocks represent *L. albus* chromosomes, green blocks represent *A. thaliana* chromosomes, brown represent *G.max,* and blue blocks represent *L. angustifolius* chromosomes.

**Additional file 6: **Duplication of ABCG subfamily and Ka/Ks ratios of *L. albus* and *A. thaliana*

**Additional file 7: **ABCG subfamily whole genome duplication and Ka/Ks ratios of *L. albus* and *G.max*

**Additional file 8: **ABCG subfamily whole genome duplication and Ka/Ks ratios of *L. albus* and *L. angustifolius*

**Additional file 9: **Collinearity relationship of the ABCG subfamily members of *L. albus* compared with *A. thaliana, G. max, L. angustifolius,* and *P. vulgaris.* Background shaded gray lines indicate the collinearity blocks of *L. albus* genome with targeted plant genome, while red lines indicate the syntenic pairs of only ABCG transporters gene subfamily between two genomes.

**Additional file 10:.** Raw data for tissue specific expression of LaABCG subfamily under phosphorus sufficient (CK) and low phosphorus (LP) condition

**Additional file 11:.** Expression pattern of ABCG subfamily members among different plant tissues under control (CK: phosphorus sufficient) and low phosphorus (LP) conditions. Fragment per kilobase of exon model per million mapped read (FPKM) values were used to visualize the gene expression on the heat map using R statistical online tool using RColorBrewer package. All the values are log2 transformed. LP-L/CK-L; leaf, CK-S/LP-S; stem, LP-RT; root tip, LP-CR; cluster root, and CK-R; root.

**Additional file 12:.** Primers used for qPCR

## Data Availability

All data generated or analyzed during this study are included in this published article [and its supplementary information files]. However, RNAseq data used in this study is available at NCBI GEO (Gene expression omnibus) repository database under GSE31132 series number at this link (https://www.ncbi.nlm.nih.gov/geo/query/acc.cgi?acc=GSE31132).
