# Supplementary material for: Identification of ABC transporter G subfamily in white lupin and functional characterization of L.albABGC29 in phosphorus use
Source: BMC Genomics. 2021 Oct 6;22:723. doi: 10.1186/s12864-021-08015-0 (PMC8495970; doi:10.1186/s12864-021-08015-0)
Supplement: Supplementary file 1 — Additional file 1:. Chromosomal distribution of White lupin ABCG transporter subfamily. Names of chromosomes are mentioned on left side, genes linked with purple line indicates tandem duplication, and the chromosome size scale is in Mb. Different colours inside the chromosome bar represent genes density of each chromosome. [file 12864_2021_8015_MOESM1_ESM.docx]

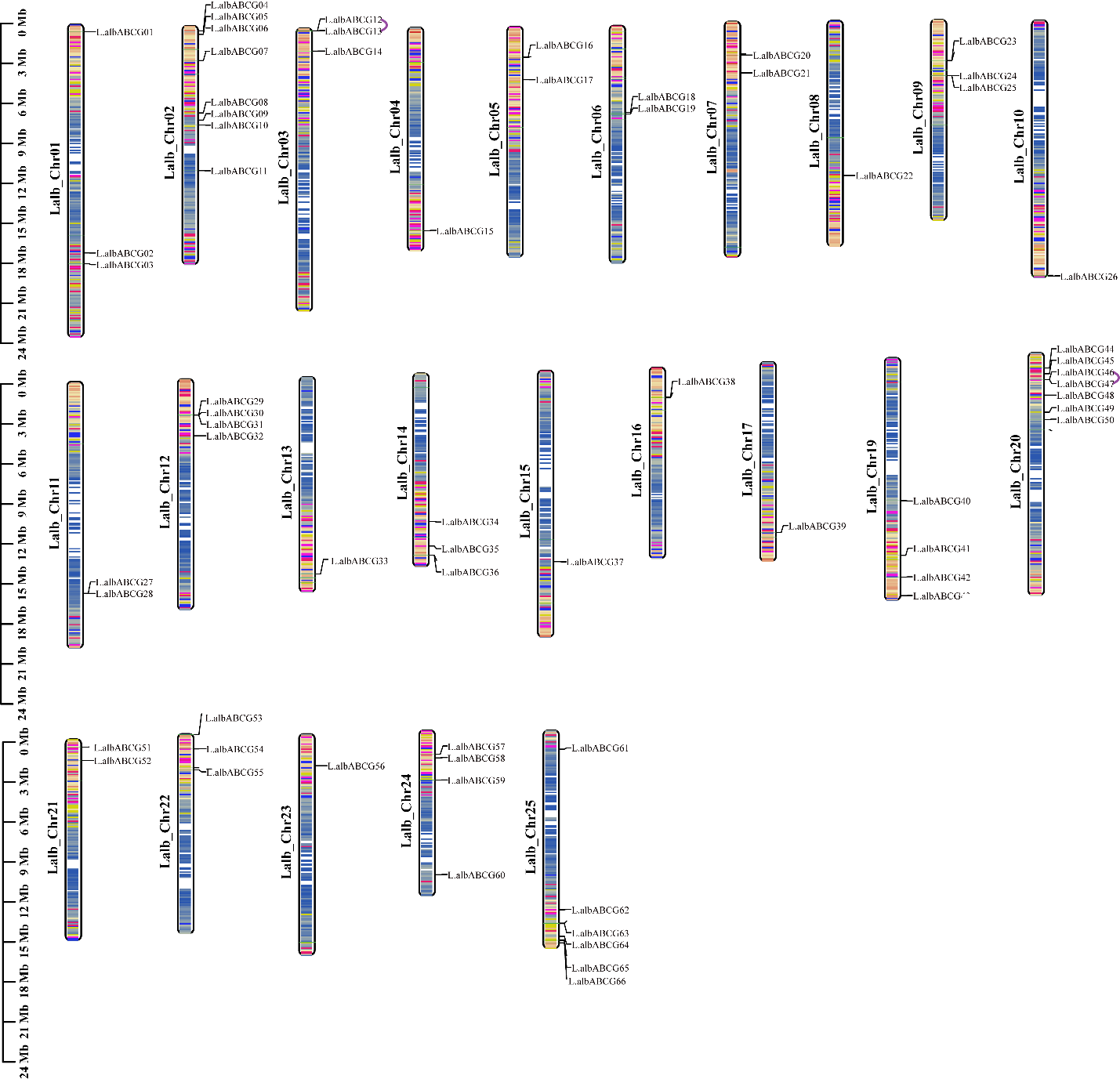


**Additional file 1** Chromosomal distribution of White lupin ABCG transporter subfamily. Names of chromosomes are mentioned on left side, genes linked with purple line indicates tandem duplication, and the chromosome size scale is in Mb. Different colours inside the chromosome bar represent genes density of each chromosome.
