# Supplementary material for: Identification of ABC transporter G subfamily in white lupin and functional characterization of L.albABGC29 in phosphorus use
Source: BMC Genomics. 2021 Oct 6;22:723. doi: 10.1186/s12864-021-08015-0 (PMC8495970; doi:10.1186/s12864-021-08015-0)
Supplement: Supplementary file 2 — Additional file 2: Detailed information of L. albus ABCG subfamily genes [file 12864_2021_8015_MOESM2_ESM.doc]

| **Additional file 2. Detailed information of *L. albus* ABCG subfamily genes** | | | | | |  |  |  |  |  |  |  |
| --- | --- | --- | --- | --- | --- | --- | --- | --- | --- | --- | --- | --- |
| **La ID** | **Renamed ID** | **Chr_no.** | **Gene start** | **Gene end** | **Gene length** | **Protein length** | **Exon** | **Intron** | **Domain** | **Sub-family** | **Subcellular location** | **At homolog** |
| Lalb_Chr01g0001061 | *L.albABCG01* | Chr_01 | 555727 | 560497 | 4771 | 683 | 9 | 8 | NBD-TMD | G half | Plasma membrane | AT3G21090 |
| Lalb_Chr01g0016911 | *L.albABCG02* | Chr_01 | 17156903 | 17160432 | 3530 | 660 | 4 | 3 | NBD-TMD | G half | Plasma membrane | AT4G27420 |
| Lalb_Chr01g0017721 | *L.albABCG03* | Chr_01 | 17960428 | 17970753 | 10326 | 737 | 10 | 9 | NBD-TMD | G half | Plasma membrane | AT5G06530 |
| Lalb_Chr02g0141291 | *L.albABCG04* | Chr_02 | 371985 | 374379 | 2395 | 537 | 3 | 2 | NBD-TMD-TMD | G half | Plasma membrane | AT5G52860 |
| Lalb_Chr02g0141771 | *L.albABCG05* | Chr_02 | 637387 | 641111 | 3725 | 439 | 2 | 1 | NBD | G half | Mitochondria | AT1G71960 |
| Lalb_Chr02g0143881 | *L.albABCG06* | Chr_02 | 1733412 | 1736023 | 2612 | 730 | 1 | 0 | NBD-TMD | G half | Plasma membrane | AT2G39350 |
| Lalb_Chr02g0145491 | *L.albABCG07* | Chr_02 | 2601264 | 2609424 | 8161 | 729 | 11 | 10 | NBD-TMD | G half | Plasma membrane | AT2G01320 |
| Lalb_Chr02g0151441 | *L.albABCG08* | Chr_02 | 6530293 | 6541058 | 10766 | 1462 | 20 | 19 | NBD-NBD-TMD-NBD-TMD | G full | Plasma membrane | AT2G36380 |
| Lalb_Chr02g0151881 | *L.albABCG09* | Chr_02 | 7066876 | 7071523 | 4648 | 676 | 5 | 4 | NBD-TMD | G half | Plasma membrane | AT3G25620 |
| Lalb_Chr02g0152231 | *L.albABCG10* | Chr_02 | 7437852 | 7441953 | 4102 | 822 | 6 | 5 | NBD-TMD | G half | Plasma membrane | AT5G60740 |
| Lalb_Chr02g0154221 | *L.albABCG11* | Chr_02 | 10855480 | 10868421 | 12942 | 745 | 10 | 9 | NBD-TMD | G half | Plasma membrane | AT5G06530 |
| Lalb_Chr03g0024491 | *L.**albABCG12* | Chr_03 | 187484 | 191451 | 3968 | 529 | 7 | 6 | NBD-TMD | G half | Plasma membrane | AT3G21090 |
| Lalb_Chr03g0024501 | *L.albABCG13* | Chr_03 | 192874 | 199792 | 6919 | 684 | 9 | 8 | NBD-TMD | G half | Plasma membrane | AT3G21090 |
| Lalb_Chr03g0027541 | *L.albABCG14* | Chr_03 | 1710638 | 1717118 | 6481 | 995 | 12 | 11 | NBD | G half | Plasma membrane | AT5G60740 |
| Lalb_Chr04g0262571 | *L.albABCG15* | Chr_04 | 15246174 | 15254025 | 7852 | 1425 | 22 | 21 | NBD-NBD-TMD-NBD-TMD | G full | Plasma membrane | AT1G15520 |
| Lalb_Chr05g0215061 | *L.albABCG16* | Chr_05 | 2288472 | 2291756 | 3285 | 644 | 4 | 3 | NBD-TMD | G half | Plasma membrane | AT1G31770 |
| Lalb_Chr05g0217901 | *L.albABCG17* | Chr_05 | 3973126 | 3975462 | 2337 | 650 | 1 | 0 | NBD-TMD | G half | Plasma membrane | AT5G19410 |
| Lalb_Chr06g0170281 | *L.albABCG18* | Chr_06 | 6502768 | 6508502 | 5735 | 679 | 5 | 4 | NBD-TMD | G half | Plasma membrane | AT3G25620 |
| Lalb_Chr06g0170361 | *L.albABCG19* | Chr_06 | 6630311 | 6639906 | 9596 | 1119 | 14 | 13 | NBD | G half | Plasma membrane | AT1G53390 |
| Lalb_Chr07g0181771 | *L.albABCG20* | Chr_07 | 2502170 | 2514865 | 12696 | 1407 | 24 | 23 | NBD-NBD-TMD-NBD-TMD | G full | Plasma membrane | AT3G53480 |
| Lalb_Chr07g0184361 | *L.albABCG21* | Chr_07 | 3845852 | 3848329 | 2478 | 643 | 1 | 0 | NBD-TMD | G half | Plasma membrane | AT2G13610 |
| Lalb_Chr08g0238251 | *L.albABCG22* | Chr_08 | 11608138 | 11608708 | 571 | 130 | 1 | 0 | NBD | G half | Nucleus | AT2G13610 |
| Lalb_Chr09g0324981 | *L.albABCG23* | Chr_09 | 3041603 | 3044543 | 2941 | 642 | 4 | 3 | NBD-TMD | G half | Plasma membrane | AT1G31770 |
| Lalb_Chr09g0326821 | *L.albABCG24* | Chr_09 | 4179958 | 4185178 | 5221 | 660 | 8 | 7 | NBD-TMD | G half | Plasma membrane | AT1G17840 |
| Lalb_Chr09g0326841 | *L.albABCG25* | Chr_09 | 4186518 | 4188986 | 2469 | 457 | 7 | 6 | NBD-TMD | G half | Golgi appratus | AT1G17840 |
| Lalb_Chr10g0107501 | *L.albABCG26* | Chr_10 | 19193795 | 19197442 | 3648 | 693 | 9 | 8 | NBD-TMD | G half | Plasma membrane | AT3G13220 |
| Lalb_Chr11g0071971 | *L.albABCG27* | Chr_11 | 15885093 | 15887804 | 2712 | 653 | 8 | 7 | NBD-TMD | G half | Plasma membrane | AT3G30842 |
| Lalb_Chr11g0071981 | *L.albABCG28* | Chr_11 | 15888060 | 15892786 | 4727 | 609 | 11 | 10 | NBD-TMD | G half | Plasma membrane | AT3G30842 |
| Lalb_Chr12g0200641 | *L.albABCG29* | Chr_12 | 2683607 | 2692917 | 9311 | 1453 | 24 | 23 | NBD-NBD-TMD-NBD-TMD | G full | Plasma membrane | AT3G53480 |
| Lalb_Chr12g0200661 | *L.albABCG30* | Chr_12 | 2693727 | 2699728 | 6002 | 1094 | 18 | 17 | TMD-NBD-TMD | G half | Plasma membrane | AT3G53480 |
| Lalb_Chr12g0200671 | *L.albABCG31* | Chr_12 | 2699730 | 2701622 | 1893 | 214 | 4 | 3 | NBD-NBD | G half | Cytoplam | AT2G37280 |
| Lalb_Chr12g0203121 | *L.albABCG32* | Chr_12 | 4252198 | 4254468 | 2271 | 632 | 1 | 0 | NBD-TMD | G half | Plasma membrane | AT2G13610 |
| Lalb_Chr13g0302241 | *L.albABCG33* | Chr_13 | 14760548 | 14769788 | 9241 | 1418 | 24 | 23 | NBD-NBD-TMD-NBD-TMD | G full | Plasma membrane | AT2G26910 |
| Lalb_Chr14g0370021 | *L.albABCG34* | Chr_14 | 11108624 | 11110873 | 2250 | 244 | 2 | 1 | NBD | G half | Golgi appratus | AT1G17840 |
| Lalb_Chr14g0373051 | *L.albABCG35* | Chr_14 | 12939999 | 12949467 | 9469 | 1489 | 22 | 21 | NBD-NBD-TMD-NBD-TMD | G full | Plasma membrane | AT1G59870 |
| Lalb_Chr14g0374501 | *L.albABCG36* | Chr_14 | 13624340 | 13629870 | 5531 | 723 | 11 | 10 | NBD-TMD | G half | Plasma membrane | AT2G28070 |
| Lalb_Chr15g0082891 | *L.albABCG37* | Chr_15 | 14332615 | 14339705 | 7091 | 1248 | 19 | 18 | NBD-TMD-NBD-TMD | G full | Plasma membrane | AT2G29940 |
| Lalb_Chr16g0380341 | *L.albABCG38* | Chr_16 | 2236952 | 2240080 | 3129 | 645 | 4 | 3 | NBD-TMD-TMD | G half | Plasma membrane | AT1G31770 |
| Lalb_Chr17g0345071 | *L.albABCG39* | Chr_17 | 12788200 | 12800962 | 12763 | 1455 | 24 | 23 | NBD-NBD-TMD-NBD-TMD | G full | Plasma membrane | AT2G29940 |
| Lalb_Chr19g0129251 | *L.albABCG40* | Chr_19 | 10707850 | 10719479 | 11630 | 1454 | 20 | 19 | NBD-NBD-TMD-NBD-TMD | G full | Plasma membrane | AT1G66950 |
| Lalb_Chr19g0134321 | *L.albABCG41* | Chr_19 | 14813570 | 14817065 | 3496 | 650 | 4 | 3 | NBD-TMD | G half | Plasma membrane | AT4G27420 |
| Lalb_Chr19g0137101 | *L.albABCG42* | Chr_19 | 16451418 | 16457527 | 6110 | 1120 | 13 | 12 | NBD | G half | Plasma membrane | AT5G60740 |
| Lalb_Chr19g0139771 | *L.albABCG43* | Chr_19 | 17837929 | 17843435 | 5507 | 724 | 9 | 8 | NBD-TMD | G half | Plasma membrane | AT3G21090 |
| Lalb_Chr20g0109361 | *L.albABCG44* | Chr_20 | 1145650 | 1147488 | 1839 | 612 | 1 | 0 | NBD-TMD | G half | Plasma membrane | AT1G53270 |
| Lalb_Chr20g0109911 | *L.albABCG45* | Chr_20 | 1575613 | 1578036 | 2424 | 632 | 4 | 3 | NBD-TMD | G half | Ensoplasmic reticulum | AT4G27420 |
| Lalb_Chr20g0110571 | *L.albABCG46* | Chr_20 | 2008372 | 2013802 | 5431 | 676 | 9 | 8 | NBD-TMD | G half | Plasma membrane | AT3G21090 |
| Lalb_Chr20g0110581 | *L.albABCG47* | Chr_20 | 2015716 | 2020500 | 4785 | 661 | 9 | 8 | NBD-TMD | G half | Plasma membrane | AT3G21090 |
| Lalb_Chr20g0112091 | *L.albABCG48* | Chr_20 | 3171567 | 3179295 | 7729 | 1478 | 19 | 18 | NBD-NBD-TMD-NBD-TMD | G full | Plasma membrane | AT1G66950 |
| Lalb_Chr20g0113531 | *L.albABCG49* | Chr_20 | 4455961 | 4464275 | 8315 | 1295 | 22 | 21 | NBD-NBD-TMD-NBD-TMD | G full | Plasma membrane | AT1G15520 |
| Lalb_Chr20g0114061 | *L.albABCG50* | Chr_20 | 5010996 | 5015827 | 4832 | 679 | 9 | 8 | NBD-TMD | G half | Plasma membrane | AT3G13220 |
| Lalb_Chr21g0305731 | *L.albABCG51* | Chr_21 | 594481 | 599541 | 5061 | 725 | 3 | 2 | NBD-TMD | G half | Plasma membrane | AT1G71960 |
| Lalb_Chr21g0307171 | *L.albABCG52* | Chr_21 | 1588379 | 1590869 | 2491 | 747 | 1 | 0 | NBD-TMD | G half | Plasma membrane | AT2G39350 |
| Lalb_Chr22g0350341 | *L.albABCG53* | Chr_22 | 534743 | 541601 | 6859 | 723 | 11 | 10 | NBD-TMD | G half | Plasma membrane | AT2G28070 |
| Lalb_Chr22g0351531 | *L.albABCG54* | Chr_22 | 1113771 | 1123618 | 9848 | 1488 | 22 | 21 | NBD-NBD-TMD-NBD-TMD | G full | Plasma membrane | AT1G59870 |
| Lalb_Chr22g0354141 | *L.albABCG55* | Chr_22 | 2682682 | 2688543 | 5862 | 707 | 10 | 9 | NBD-TMD | G half | Plasma membrane | AT1G17840 |
| Lalb_Chr23g0268841 | *L.albABCG56* | Chr_23 | 2367684 | 2375314 | 7631 | 1444 | 22 | 21 | NBD-NBD-TMD-TMD-NBD-TMD | G full | Plasma membrane | AT1G15520 |
| Lalb_Chr24g0395671 | *L.albABCG57* | Chr_24 | 1794623 | 1801898 | 7276 | 1426 | 21 | 20 | NBD-NBD-TMD-NBD-TMD | G full | Plasma membrane | AT1G15520 |
| Lalb_Chr24g0396141 | *L.albABCG58* | Chr_24 | 2057163 | 2067347 | 10185 | 1410 | 20 | 19 | NBD-NBD-TMD-NBD-TMD | G full | Plasma membrane | AT1G59870 |
| Lalb_Chr24g0398651 | *L.albABCG59* | Chr_24 | 3720421 | 3728429 | 8009 | 708 | 10 | 9 | NBD-TMD | G half | Plasma membrane | AT1G17840 |
| Lalb_Chr24g0402281 | *L.albABCG60* | Chr_24 | 10821206 | 10830463 | 9258 | 1443 | 23 | 22 | NBD-NBD-TMD-NBD-TMD | G full | Plasma membrane | AT1G15520 |
| Lalb_Chr25g0280151 | *L.albABCG61* | Chr_25 | 1396291 | 1409247 | 12957 | 1487 | 22 | 21 | NBD-NBD-TMD-NBD-TMD | G full | Plasma membrane | AT1G59870 |
| Lalb_Chr25g0285971 | *L.albABCG62* | Chr_25 | 13445416 | 13447239 | 1824 | 607 | 1 | 0 | NBD-TMD | G half | Plasma membrane | AT1G53270 |
| Lalb_Chr25g0287201 | *L.albABCG63* | Chr_25 | 14482153 | 14485250 | 3098 | 295 | 4 | 3 | NBD | G half | Cytoplasm | AT3G21090 |
| Lalb_Chr25g0288571 | *L.albABCG64* | Chr_25 | 15433052 | 15439197 | 6146 | 1327 | 14 | 13 | TMD-NBD-TMD | G half | Plasma membrane | AT1G66950 |
| Lalb_Chr25g0289051 | *L.albABCG65* | Chr_25 | 15750339 | 15759223 | 8885 | 1108 | 14 | 13 | NBD | G half | Plasma membrane | AT2G37010 |
| Lalb_Chr25g0289371 | *L.albABCG66* | Chr_25 | 15927522 | 15930335 | 2814 | 737 | 2 | 1 | NBD-TMD | G half | Plasma membrane | AT2G37360 |
