# Supplementary material for: Identification of ABC transporter G subfamily in white lupin and functional characterization of L.albABGC29 in phosphorus use
Source: BMC Genomics. 2021 Oct 6;22:723. doi: 10.1186/s12864-021-08015-0 (PMC8495970; doi:10.1186/s12864-021-08015-0)
Supplement: Supplementary file 3 — Additional file 3:. Conserved protein sequences of ten motifs identifed in ABCG subfamily of white lupin. Motif scan and sequence logos were generated in linux based MEME program. [file 12864_2021_8015_MOESM3_ESM.docx]

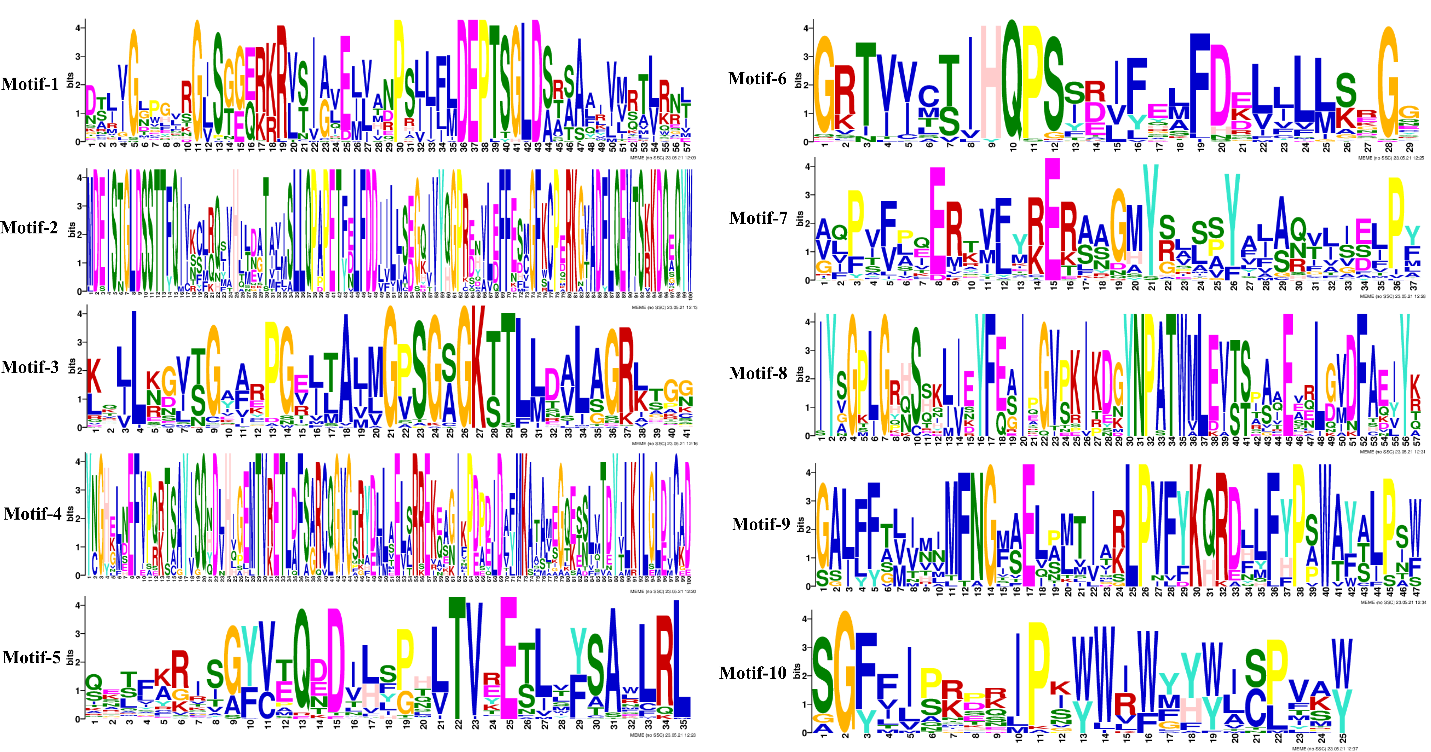


**Additional file 3** Conserved protein sequences of ten motifs identifed in ABCG subfamily of white lupin. Motif scan and sequence logos were generated in linux based MEME program.
