# Supplementary material for: Identification of ABC transporter G subfamily in white lupin and functional characterization of L.albABGC29 in phosphorus use
Source: BMC Genomics. 2021 Oct 6;22:723. doi: 10.1186/s12864-021-08015-0 (PMC8495970; doi:10.1186/s12864-021-08015-0)
Supplement: Supplementary file 4 — Additional file 4: Whole genome duplication of ABCG subfamily and Ka/Ks ratios in the L. albus genome [file 12864_2021_8015_MOESM4_ESM.doc]

**Additional file 4. Whole genome duplication of ABCG subfamily and Ka/Ks ratios in the *L. albus* genome**

| **Seq_1** | **Seq_2** | **Ka** | **Ks** | **Ka/Ks** | **Type** |
| --- | --- | --- | --- | --- | --- |
| Lalb_Chr01g0017721 | Lalb_Chr02g0154221 | 0.101 | 0.604 | 0.167 | Segmental duplication |
| Lalb_Chr01g0001061 | Lalb_Chr03g0024491 | 0.202 | 1.491 | 0.135 | Segmental duplication |
| Lalb_Chr03g0024491 | Lalb_Chr03g0024501 | 0.205 | 1.523 | 0.135 | Tandem duplication |
| Lalb_Chr02g0151881 | Lalb_Chr06g0170281 | 0.064 | 0.352 | 0.183 | Segmental duplication |
| Lalb_Chr07g0184361 | Lalb_Chr08g0238251 | 0.162 | 0.497 | 0.327 | Segmental duplication |
| Lalb_Chr05g0215061 | Lalb_Chr09g0324981 | 0.049 | 0.263 | 0.187 | Segmental duplication |
| Lalb_Chr07g0181771 | Lalb_Chr12g0200641 | 0.262 | 1.488 | 0.176 | Segmental duplication |
| Lalb_Chr07g0184361 | Lalb_Chr12g0203121 | 0.063 | 0.203 | 0.311 | Segmental duplication |
| Lalb_Chr07g0184361 | Lalb_Chr12g0203121 | 0.063 | 0.203 | 0.311 | Segmental duplication |
| Lalb_Chr08g0238251 | Lalb_Chr12g0203121 | 0.164 | 0.532 | 0.309 | Segmental duplication |
| Lalb_Chr05g0215061 | Lalb_Chr16g0380341 | 0.06 | 0.198 | 0.301 | Segmental duplication |
| Lalb_Chr09g0324981 | Lalb_Chr16g0380341 | 0.057 | 0.203 | 0.281 | Segmental duplication |
| Lalb_Chr03g0027541 | Lalb_Chr19g0137101 | 0.026 | 0.27 | 0.098 | Segmental duplication |
| Lalb_Chr01g0001061 | Lalb_Chr19g0139771 | 0.22 | 1.38 | 0.16 | Segmental duplication |
| Lalb_Chr03g0024491 | Lalb_Chr19g0139771 | 0.056 | 0.214 | 0.262 | Segmental duplication |
| Lalb_Chr19g0134321 | Lalb_Chr20g0109911 | 0.167 | 0.632 | 0.264 | Segmental duplication |
| Lalb_Chr20g0110571 | Lalb_Chr20g0110581 | 0.359 | 1.437 | 0.25 | Tandem duplication |
| Lalb_Chr02g0143881 | Lalb_Chr21g0307171 | 0.056 | 0.551 | 0.101 | Segmental duplication |
| Lalb_Chr14g0374501 | Lalb_Chr22g0350341 | 0.041 | 0.285 | 0.142 | Segmental duplication |
| Lalb_Chr14g0373051 | Lalb_Chr22g0351531 | 0.03 | 0.229 | 0.132 | Segmental duplication |
| Lalb_Chr14g0373051 | Lalb_Chr22g0351531 | 0.03 | 0.229 | 0.132 | Segmental duplication |
| Lalb_Chr04g0262571 | Lalb_Chr24g0395671 | 0.106 | 0.584 | 0.182 | Segmental duplication |
| Lalb_Chr14g0373051 | Lalb_Chr24g0396141 | 0.04 | 0.262 | 0.153 | Segmental duplication |
| Lalb_Chr22g0351531 | Lalb_Chr24g0396141 | 0.041 | 0.251 | 0.164 | Segmental duplication |
| Lalb_Chr22g0354141 | Lalb_Chr24g0398651 | 0.02 | 0.367 | 0.054 | Segmental duplication |
| Lalb_Chr23g0268841 | Lalb_Chr24g0402281 | 0.175 | 1.271 | 0.138 | Segmental duplication |
| Lalb_Chr14g0373051 | Lalb_Chr25g0280151 | 0.117 | 0.628 | 0.187 | Segmental duplication |
| Lalb_Chr22g0351531 | Lalb_Chr25g0280151 | 0.117 | 0.603 | 0.194 | Segmental duplication |
| Lalb_Chr24g0396141 | Lalb_Chr25g0280151 | 0.127 | 0.635 | 0.2 | Segmental duplication |
| Lalb_Chr20g0109361 | Lalb_Chr25g0285971 | 0.045 | 0.272 | 0.165 | Segmental duplication |
| Lalb_Chr20g0112091 | Lalb_Chr25g0288561 | 0.219 | 0.49 | 0.446 | Segmental duplication |
| Lalb_Chr19g0137101 | Lalb_Chr25g0289051 | 0.268 | 1.6 | 0.167 | Segmental duplication |
