# Supplementary material for: Identification of ABC transporter G subfamily in white lupin and functional characterization of L.albABGC29 in phosphorus use
Source: BMC Genomics. 2021 Oct 6;22:723. doi: 10.1186/s12864-021-08015-0 (PMC8495970; doi:10.1186/s12864-021-08015-0)
Supplement: Supplementary file 5 — Additional file 5: Duplication gene pairs identified with Synteny analysis in L. albus ATPase-binding cassette transporters (ABC) gene family. a duplicated gene pairs in L. albus vs A. thaliana b duplicated gene pairs in L. albus vs G. max. c duplicated gene pairs in L. albus vs L. angustifolius. Different color lines exhibited paralogous pairs of ABC transporter genes and their subsequent location on chromosomes. The shaded gray background shows the synteny pairs of the whole-genome and respective chromosome numbers are labeled outside the circle. Pink blocks represent L. albus chromosomes, green blocks represent A. thaliana chromosomes, brown represent G.max, and blue blocks represent L. angustifolius chromosomes. [file 12864_2021_8015_MOESM5_ESM.docx]

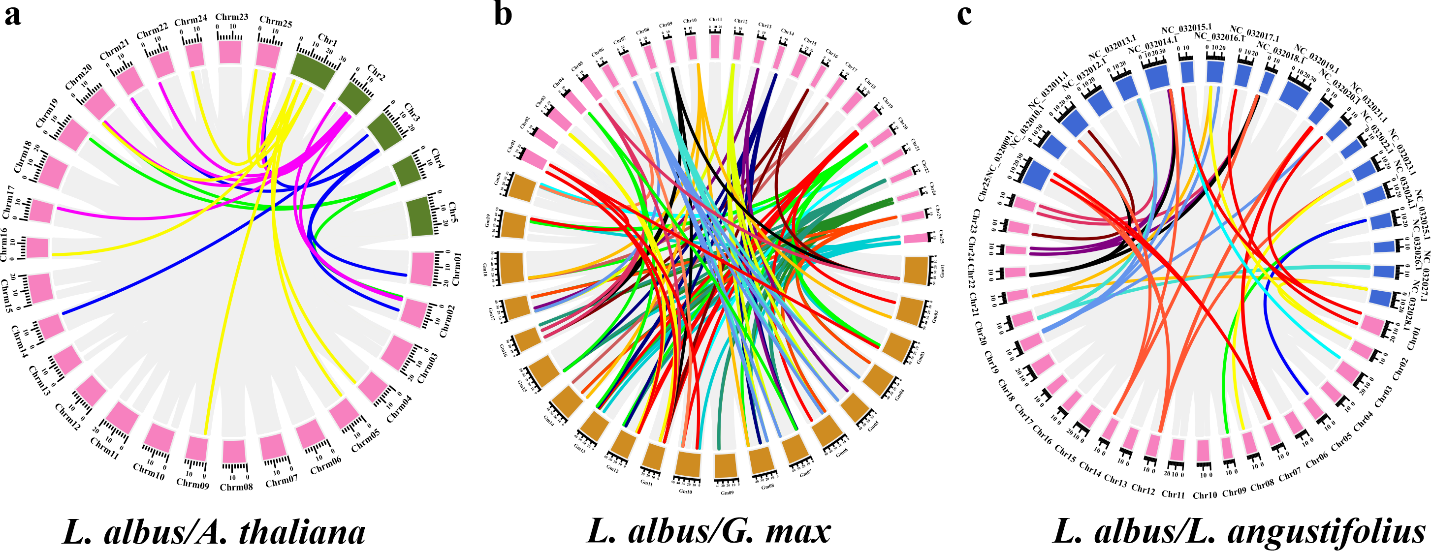


**Additional file 5.** Duplication gene pairs identified with Synteny analysis in *L. albus* ATPase-binding cassette transporters (ABC) gene family. **a** duplicated gene pairs in *L. albus* vs *A. thaliana* **b** duplicated gene pairs in *L. albus* vs *G. max*. **c** duplicated gene pairs in *L. albus* vs *L. angustifolius*. Different color lines exhibited paralogous pairs of ABC transporter genes and their subsequent location on chromosomes. The shaded gray background shows the synteny pairs of the whole-genome and respective chromosome numbers are labeled outside the circle. Pink blocks represent *L. albus* chromosomes, green blocks represent *A. thaliana* chromosomes, brown represent *G.max,* and blue blocks represent *L*. *angustifolius* chromosomes.
