# Supplementary material for: Identification of ABC transporter G subfamily in white lupin and functional characterization of L.albABGC29 in phosphorus use
Source: BMC Genomics. 2021 Oct 6;22:723. doi: 10.1186/s12864-021-08015-0 (PMC8495970; doi:10.1186/s12864-021-08015-0)
Supplement: Supplementary file 6 — Additional file 6: Duplication of ABCG subfamily and Ka/Ks ratios of L. albus and A. thaliana [file 12864_2021_8015_MOESM6_ESM.doc]

**Additional file 6. Duplication of ABCG subfamily and Ka/Ks ratios of *L. albus* and *A. thaliana***

| **Seq_1** | **Seq_2** | **Ka** | **Ks** | **Ka_Ks** | **Type** |
| --- | --- | --- | --- | --- | --- |
| Lalb_Chr04g0262571 | AT1G15520.1 | 0.193 | 2.508 | 0.077 | Segmental duplication |
| Lalb_Chr24g0395671 | AT1G15520.1 | 0.194 | 2.386 | 0.081 | Segmental duplication |
| Lalb_Chr05g0215061 | AT1G31770.1 | 0.160 | 1.684 | 0.095 | Segmental duplication |
| Lalb_Chr09g0324981 | AT1G31770.1 | 0.157 | 1.580 | 0.100 | Segmental duplication |
| Lalb_Chr16g0380341 | AT1G31770.1 | 0.170 | 1.561 | 0.109 | Segmental duplication |
| Lalb_Chr20g0109361 | AT1G53270.1 | 0.280 | 1.232 | 0.191 | Segmental duplication |
| Lalb_Chr25g0285971 | AT1G53270.1 | 0.276 | 1.965 | 0.141 | Segmental duplication |
| Lalb_Chr02g0145491 | AT2G01320.3 | 0.171 | 1.754 | 0.098 | Segmental duplication |
| Lalb_Chr22g0350341 | AT2G28070.1 | 0.141 | 2.277 | 0.062 | Segmental duplication |
| Lalb_Chr17g0345071 | AT2G29940.1 | 0.189 | 1.953 | 0.097 | Segmental duplication |
| Lalb_Chr20g0112091 | AT2G36380.1 | 0.237 | 1.691 | 0.140 | Segmental duplication |
| Lalb_Chr25g0289051 | AT2G37010.1 | 0.244 | 1.806 | 0.135 | Segmental duplication |
| Lalb_Chr25g0289371 | AT2G37360.1 | 0.244 | 2.325 | 0.105 | Segmental duplication |
| Lalb_Chr02g0143881 | AT2G39350.1 | 0.202 | 1.215 | 0.175 | Segmental duplication |
| Lalb_Chr21g0307171 | AT2G39350.1 | 0.199 | 1.355 | 0.150 | Segmental duplication |
| Lalb_Chr25g0289371 | AT3G53510.1 | 0.213 | 2.356 | 0.090 | Segmental duplication |
| Lalb_Chr02g0143881 | AT3G55090.1 | 0.210 | 1.655 | 0.127 | Segmental duplication |
| Lalb_Chr21g0307171 | AT3G55090.1 | 0.183 | 3.255 | 0.056 | Segmental duplication |
| Lalb_Chr02g0141291 | AT4G25750.1 | 0.249 | 2.718 | 0.092 | Segmental duplication |
| Lalb_Chr19g0134321 | AT4G27420.1 | 0.313 | 3.291 | 0.095 | Segmental duplication |
| Lalb_Chr20g0109911 | AT4G27420.1 | 0.366 | 4.738 | 0.077 | Segmental duplication |
| Lalb_Chr02g0154221 | AT5G06530.1 | 0.160 | 3.050 | 0.052 | Segmental duplication |
| Lalb_Chr02g0141291 | AT5G52860.1 | 0.239 | 1.490 | 0.195 | Segmental duplication |
| Lalb_Chr03g0027541 | AT5G60740.1 | 0.171 | 1.808 | 0.094 | Segmental duplication |
| Lalb_Chr19g0137101 | AT5G60740.1 | 0.208 | 1.703 | 0.122 | Segmental duplication |
