# Supplementary material for: Identification of ABC transporter G subfamily in white lupin and functional characterization of L.albABGC29 in phosphorus use
Source: BMC Genomics. 2021 Oct 6;22:723. doi: 10.1186/s12864-021-08015-0 (PMC8495970; doi:10.1186/s12864-021-08015-0)
Supplement: Supplementary file 7 — Additional file 7: ABCG subfamily whole genome duplication and Ka/Ks ratios of L. albus and G.max [file 12864_2021_8015_MOESM7_ESM.doc]

**Additional file 7. ABCG subfamily whole genome duplication and Ka/Ks ratios of *L. albus* and *G.max***

| **Seq_1** | **Seq_2** | **Ka** | **Ks** | **Ka/Ks** | **Type** |
| --- | --- | --- | --- | --- | --- |
| Lalb_Chr02g0151881 | Glyma01g22850 | 0.14 | 0.717 | 0.196 | Segmental duplication |
| Lalb_Chr01g0001061 | Glyma03g34080 | 0.952 | 2.179 | 0.437 | Segmental duplication |
| Lalb_Chr02g0151441 | Glyma03g35030 | 0.246 | 1.64 | 0.15 | Segmental duplication |
| Lalb_Chr01g0017721 | Glyma03g36310 | 0.096 | 0.432 | 0.222 | Segmental duplication |
| Lalb_Chr02g0154221 | Glyma03g36310 | 0.099 | 0.507 | 0.195 | Segmental duplication |
| Lalb_Chr04g0262571 | Glyma07g03780 | 0.076 | 0.453 | 0.169 | Segmental duplication |
| Lalb_Chr01g0017721 | Glyma10g11000 | 0.09 | 0.607 | 0.148 | Segmental duplication |
| Lalb_Chr02g0154221 | Glyma10g11000 | 0.061 | 0.493 | 0.125 | Segmental duplication |
| Lalb_Chr02g0151441 | Glyma10g34700 | 0.108 | 0.331 | 0.327 | Segmental duplication |
| Lalb_Chr02g0151881 | Glyma10g34700 | 0.916 | 2.431 | 0.377 | Segmental duplication |
| Lalb_Chr02g0151441 | Glyma10g34980 | 0.9 | 3.433 | 0.262 | Segmental duplication |
| Lalb_Chr02g0151881 | Glyma10g34980 | 0.104 | 0.707 | 0.147 | Segmental duplication |
| Lalb_Chr02g0141291 | Glyma10g37420 | 0.117 | 1.123 | 0.104 | Segmental duplication |
| Lalb_Chr02g0145491 | Glyma10g41110 | 0.075 | 0.343 | 0.218 | Segmental duplication |
| Lalb_Chr01g0001061 | Glyma11g09950 | 0.201 | 1.516 | 0.132 | Segmental duplication |
| Lalb_Chr03g0024491 | Glyma11g09950 | 0.099 | 0.412 | 0.24 | Segmental duplication |
| Lalb_Chr01g0001061 | Glyma12g02290 | 0.207 | 1.654 | 0.125 | Segmental duplication |
| Lalb_Chr03g0024491 | Glyma12g02290 | 0.099 | 0.416 | 0.238 | Segmental duplication |
| Lalb_Chr02g0141291 | Glyma13g35540 | 1.012 | 2.466 | 0.411 | Segmental duplication |
| Lalb_Chr04g0262571 | Glyma13g43860 | 0.371 | 1.14 | 0.325 | Segmental duplication |
| Lalb_Chr04g0262571 | Glyma15g01460 | 0.145 | 0.767 | 0.189 | Segmental duplication |
| Lalb_Chr02g0141291 | Glyma16g28891 | 0.895 | 3.128 | 0.286 | Segmental duplication |
| Lalb_Chr01g0001061 | Glyma19g36820 | 0.953 | 2.327 | 0.409 | Segmental duplication |
| Lalb_Chr01g0017721 | Glyma19g38970 | 0.095 | 0.546 | 0.173 | Segmental duplication |
| Lalb_Chr02g0154221 | Glyma19g38970 | 0.1 | 0.635 | 0.157 | Segmental duplication |
| Lalb_Chr02g0145491 | Glyma20g26160 | 0.078 | 0.388 | 0.2 | Segmental duplication |
| Lalb_Chr02g0141291 | Glyma20g30320 | 0.177 | 1.388 | 0.128 | Segmental duplication |
| Lalb_Chr02g0151881 | Glyma20g32580 | 0.108 | 0.725 | 0.148 | Segmental duplication |
| Lalb_Chr02g0151441 | Glyma20g32870 | 0.113 | 0.372 | 0.304 | Segmental duplication |
| Lalb_Chr02g0143881 | Glyma20g38610 | 0.082 | 1.549 | 0.053 | Segmental duplication |
