# Supplementary material for: Identification of ABC transporter G subfamily in white lupin and functional characterization of L.albABGC29 in phosphorus use
Source: BMC Genomics. 2021 Oct 6;22:723. doi: 10.1186/s12864-021-08015-0 (PMC8495970; doi:10.1186/s12864-021-08015-0)
Supplement: Supplementary file 8 — Additional file 8: ABCG subfamily whole genome duplication and Ka/Ks ratios of L. albus and L. angustifolius [file 12864_2021_8015_MOESM8_ESM.doc]

**Additional file 8. ABCG subfamily whole genome duplication and Ka/Ks ratios of *L. albus* and *L. angustifolius***

| **Seq_1** | **Seq_2** | **Ka** | **Ks** | **Ka/Ks** | **Type** |
| --- | --- | --- | --- | --- | --- |
| Lalb__Chr01g0001061 | XP_019412679.1 | 0.015 | 0.132 | 0.116 | Segmental duplication |
| Lalb__Chr19g0139771 | XP_019412679.1 | 0.217 | 1.286 | 0.169 | Segmental duplication |
| Lalb__Chr08g0238251 | XP_019416413.1 | 0.039 | 0.145 | 0.266 | Segmental duplication |
| Lalb__Chr12g0203121 | XP_019416413.1 | 0.14 | 0.494 | 0.283 | Segmental duplication |
| Lalb__Chr05g0215061 | XP_019421168.1 | 0.046 | 0.271 | 0.168 | Segmental duplication |
| Lalb__Chr09g0324981 | XP_019421168.1 | 0.017 | 0.101 | 0.165 | Segmental duplication |
| Lalb__Chr20g0112091 | XP_019424860.1 | 0.049 | 0.129 | 0.379 | Segmental duplication |
| Lalb__Chr20g0110571 | XP_019424918.1 | 0.031 | 0.122 | 0.253 | Segmental duplication |
| Lalb__Chr02g0143881 | XP_019429167.1 | 0.021 | 0.161 | 0.129 | Segmental duplication |
| Lalb__Chr21g0307171 | XP_019429167.1 | 0.052 | 0.537 | 0.096 | Segmental duplication |
| Lalb__Chr02g0151881 | XP_019434360.1 | 0.058 | 0.347 | 0.168 | Segmental duplication |
| Lalb__Chr06g0170281 | XP_019434360.1 | 0.018 | 0.126 | 0.14 | Segmental duplication |
| Lalb__Chr14g0373051 | XP_019436457.1 | 0.032 | 0.261 | 0.122 | Segmental duplication |
| Lalb__Chr22g0351531 | XP_019436457.1 | 0.01 | 0.123 | 0.082 | Segmental duplication |
| Lalb__Chr23g0268841 | XP_019437694.1 | 0.013 | 0.118 | 0.107 | Segmental duplication |
| Lalb__Chr20g0109361 | XP_019443884.1 | 0.047 | 0.27 | 0.173 | Segmental duplication |
| Lalb__Chr25g0285971 | XP_019443884.1 | 0.017 | 0.114 | 0.147 | Segmental duplication |
| Lalb__Chr19g0134321 | XP_019443966.1 | 0.171 | 0.592 | 0.289 | Segmental duplication |
| Lalb__Chr20g0109911 | XP_019443966.1 | 0.121 | 0.322 | 0.375 | Segmental duplication |
| Lalb__Chr20g0112091 | XP_019444266.1 | 0.119 | 0.316 | 0.377 | Segmental duplication |
| Lalb__Chr24g0402281 | XP_019447532.1 | 0.014 | 0.094 | 0.151 | Segmental duplication |
| Lalb__Chr24g0395671 | XP_019448107.1 | 0.005 | 0.13 | 0.041 | Segmental duplication |
| Lalb__Chr14g0373051 | XP_019448177.1 | 0.038 | 0.307 | 0.122 | Segmental duplication |
| Lalb__Chr22g0351531 | XP_019448177.1 | 0.035 | 0.288 | 0.122 | Segmental duplication |
| Lalb__Chr24g0396141 | XP_019448177.1 | 0.016 | 0.153 | 0.102 | Segmental duplication |
| Lalb__Chr03g0024491 | XP_019450893.1 | 0.033 | 0.098 | 0.341 | Segmental duplication |
| Lalb__Chr19g0139771 | XP_019450893.1 | 0.057 | 0.225 | 0.253 | Segmental duplication |
| Lalb__Chr01g0001061 | XP_019450894.1 | 0.023 | 0.248 | 0.091 | Segmental duplication |
| Lalb__Chr19g0134321 | XP_019451472.1 | 0.01 | 0.102 | 0.096 | Segmental duplication |
| Lalb__Chr02g0143881 | XP_019452549.1 | 0.055 | 0.571 | 0.096 | Segmental duplication |
| Lalb__Chr21g0307171 | XP_019452549.1 | 0.024 | 0.206 | 0.115 | Segmental duplication |
| Lalb__Chr07g0181771 | XP_019455889.1 | 0.26 | 1.531 | 0.17 | Segmental duplication |
| Lalb__Chr12g0200641 | XP_019455889.1 | 0.018 | 0.101 | 0.181 | Segmental duplication |
| Lalb__Chr01g0017721 | XP_019456691.1 | 0.023 | 0.142 | 0.16 | Segmental duplication |
| Lalb__Chr14g0373051 | XP_019458104.1 | 0.012 | 0.108 | 0.109 | Segmental duplication |
| Lalb__Chr24g0396141 | XP_019458104.1 | 0.037 | 0.264 | 0.139 | Segmental duplication |
| Lalb__Chr25g0280151 | XP_019458104.1 | 0.116 | 0.632 | 0.183 | Segmental duplication |
| Lalb__Chr22g0350341 | XP_019458827.1 | 0.032 | 0.297 | 0.109 | Segmental duplication |
| Lalb__Chr07g0184361 | XP_019462906.1 | 0.05 | 0.226 | 0.222 | Segmental duplication |
| Lalb__Chr12g0203121 | XP_019462906.1 | 0.058 | 0.225 | 0.257 | Segmental duplication |
| Lalb__Chr07g0181771 | XP_019463336.1 | 0.02 | 0.127 | 0.154 | Segmental duplication |
| Lalb__Chr12g0200641 | XP_019463336.1 | 0.255 | 1.588 | 0.16 | Segmental duplication |
| Lalb__Chr07g0184361 | XP_019463797.1 | 0.019 | 0.09 | 0.209 | Segmental duplication |
| Lalb__Chr12g0203121 | XP_019463797.1 | 0.07 | 0.2 | 0.35 | Segmental duplication |
