# Supplementary material for: Identification of ABC transporter G subfamily in white lupin and functional characterization of L.albABGC29 in phosphorus use
Source: BMC Genomics. 2021 Oct 6;22:723. doi: 10.1186/s12864-021-08015-0 (PMC8495970; doi:10.1186/s12864-021-08015-0)
Supplement: Supplementary file 9 — Additional file 9: Collinearity relationship of the ABCG subfamily members of L. albus compared with A. thaliana, G. max, L. angustifolius, and P. vulgaris. Background shaded gray lines indicate the collinearity blocks of L. albus genome with targeted plant genome, while red lines indicate the syntenic pairs of only ABCG transporters gene subfamily between two genomes. [file 12864_2021_8015_MOESM9_ESM.doc]

**
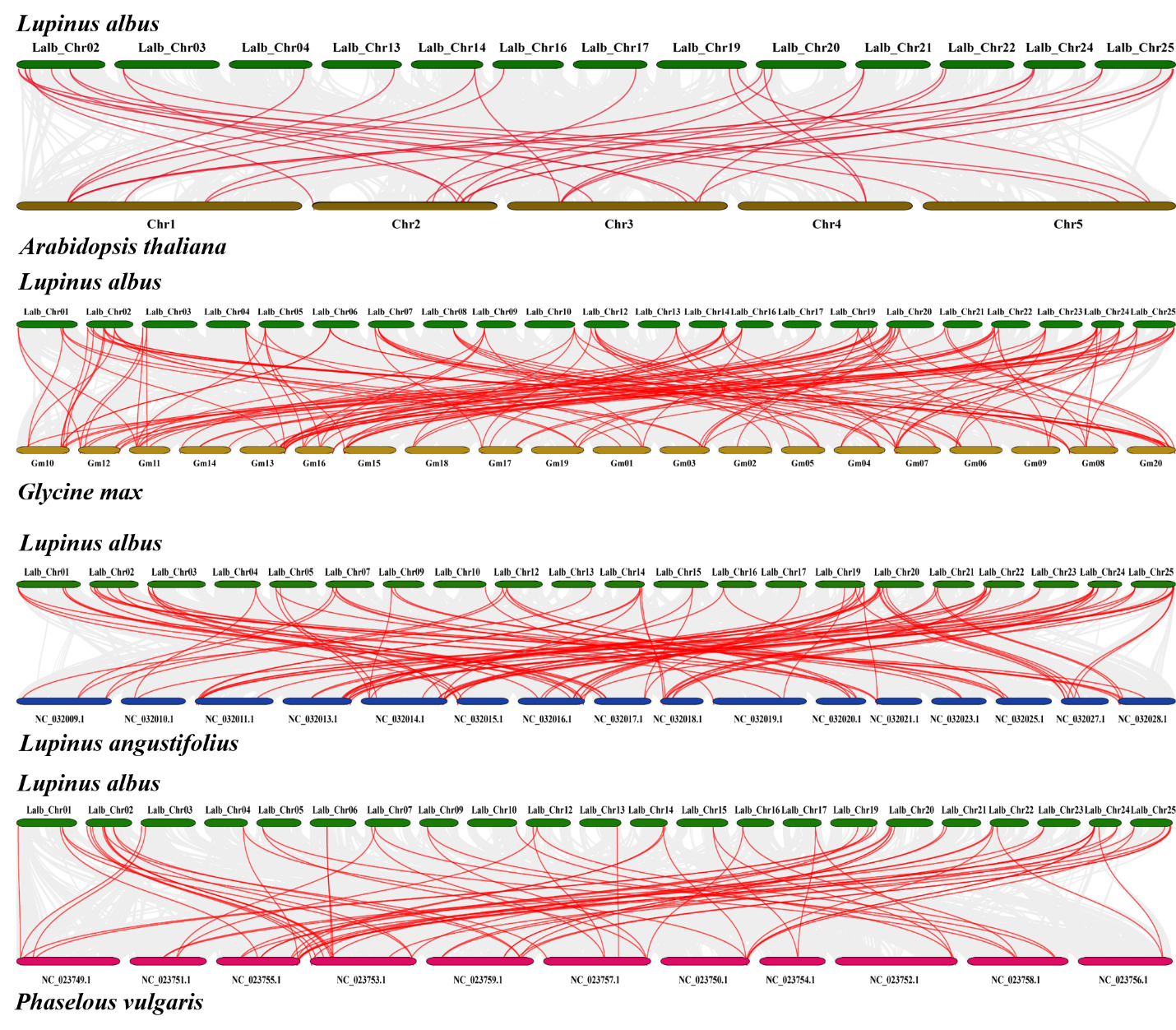
**

**Additional file 9** Collinearity relationship of the ABCG subfamily members of *L. albus* compared with *A. thaliana, G. max, L. angustifolius,* and *P. vulgaris.* Background shaded gray lines indicate the collinearity blocks of *L*. *albus* genome with targeted plant genome, while red lines indicate the syntenic pairs of only ABCG transporters gene subfamily between two genomes.
