# Supplementary material for: Identification of ABC transporter G subfamily in white lupin and functional characterization of L.albABGC29 in phosphorus use
Source: BMC Genomics. 2021 Oct 6;22:723. doi: 10.1186/s12864-021-08015-0 (PMC8495970; doi:10.1186/s12864-021-08015-0)
Supplement: Supplementary file 10 — Additional file 10:. Raw data for tissue specific expression of LaABCG subfamily under phosphorus sufficient (CK) and low phosphorus (LP) condition [file 12864_2021_8015_MOESM10_ESM.doc]

**Additional file 10 Raw data for tissue specific expression of LaABCG subfamily under phosphorus sufficient (CK) and low phosphorus (LP) condition**

| **Genes IDs** | **LP-CR** | **LP-L** | **LP-RT** | **LP-S** | **CK-L** | **CK-R** | **CK-S** |
| --- | --- | --- | --- | --- | --- | --- | --- |
| *LaABCG01* | 0.28 | 0.85 | 0.63 | 2.23 | 1.86 | 0.49 | 2.06 |
| *LaABCG02* | 0.06 | 0.12 | 0.11 | 0.2 | 0.18 | 0.02 | 0.27 |
| *LaABCG03* | 2.14 | 22.88 | 1.42 | 13.47 | 10.14 | 1.99 | 9.26 |
| *LaABCG04* | 13.8 | 0.04 | 18.58 | 13.23 | 0.02 | 21.92 | 17.16 |
| *LaABCG05* | 27.03 | 1.42 | 29.76 | 15.74 | 5.5 | 33.26 | 47.86 |
| *LaABCG06* | 0.37 | 4.78 | 0.05 | 0.46 | 8.09 | 0.04 | 0.11 |
| *LaABCG07* | 4.58 | 16.9 | 6.15 | 12.86 | 24.88 | 5.45 | 11.46 |
| *LaABCG08* | 0.33 | 9.52 | 0.12 | 2.51 | 8.07 | 0.37 | 2.04 |
| *LaABCG09* | 1.46 | 1.22 | 1.24 | 8.03 | 1.54 | 1.45 | 9.41 |
| *LaABCG10* | 1.56 | 0.04 | 0.97 | 0.07 | 0.06 | 0.88 | 0.2 |
| *LaABCG11* | 0.14 | 16.34 | 0.17 | 7.01 | 34.29 | 0.14 | 10.65 |
| *LaABCG12* | 0.94 | 12.65 | 0.66 | 10.6 | 11.9 | 0.9 | 10.96 |
| *LaABCG13* | 0.28 | 0.85 | 0.63 | 2.23 | 1.86 | 0.49 | 2.06 |
| *LaABCG14* | 0.65 | 0.46 | 1.13 | 1.44 | 1 | 0.94 | 2.23 |
| *LaABCG15* | 0.1 | 0.1 | 0.04 | 0.15 | 0.1 | 0.01 | 0.1 |
| *LaABCG16* | 2.62 | 7.55 | 6.81 | 25.83 | 16.28 | 9.12 | 15.49 |
| *LaABCG17* | 3.27 | 0.1 | 0.08 | 0.06 | 0.02 | 0.73 | 0.09 |
| *LaABCG18* | 4.02 | 4.71 | 6.51 | 18.27 | 7.2 | 6.14 | 32.24 |
| *LaABCG19* | 7.93 | 5.57 | 7.14 | 10.47 | 7.98 | 6.14 | 10.15 |
| *LaABCG20* | 29.74 | 38.25 | 14.79 | 19.84 | 18.93 | 23.58 | 17.93 |
| *LaABCG21* | 12.69 | 0.61 | 17.42 | 4.51 | 1.45 | 65.31 | 2.08 |
| *LaABCG22* | 12.69 | 0.61 | 17.42 | 4.51 | 1.45 | 65.31 | 2.08 |
| *LaABCG23* | 4.5 | 0.93 | 4.48 | 2.37 | 1.12 | 3.96 | 2.74 |
| *LaABCG24* | 0.25 | 11.26 | 0.06 | 7.44 | 28.05 | 0.11 | 1.99 |
| *LaABCG25* | 0.73 | 0.39 | 1.42 | 1.68 | 1.03 | 0.61 | 2.32 |
| *LaABCG26* | 0.11 | 0.49 | 0.07 | 0.15 | 0.19 | 0.06 | 0.13 |
| *LaABCG27* | 0.14 | 0.05 | 0.23 | 0.14 | 0.05 | 0.05 | 0.2 |
| *LaABCG28* | 0.14 | 0.05 | 0.23 | 0.14 | 0.05 | 0.05 | 0.2 |
| *LaABCG29* | 3.74 | 0.03 | 0.08 | 0.06 | 0.04 | 0.32 | 0.11 |
| *LaABCG30* | 3.5 | 0.65 | 1.38 | 1.07 | 0.33 | 3.39 | 0.84 |
| *LaABCG31* | 3.5 | 0.65 | 1.38 | 1.07 | 0.33 | 3.39 | 0.84 |
| *LaABCG32* | 2.21 | 0.3 | 2.8 | 4.73 | 2.22 | 22.08 | 4.41 |
| *LaABCG33* | 5.14 | 7.98 | 2.16 | 10.81 | 13.43 | 2.83 | 11.03 |
| *LaABCG34* | 1.66 | 7.61 | 2.03 | 4.16 | 7.41 | 1.3 | 3.38 |
| *LaABCG35* | 124.8 | 316.44 | 43.27 | 209.56 | 131.11 | 75.5 | 168.38 |
| *LaABCG36* | 2.95 | 0.7 | 7.61 | 6.21 | 3.04 | 3.17 | 6.85 |
| *LaABCG37* | 0.14 | 0.08 | 0.32 | 0.39 | 0.05 | 0.13 | 0.6 |
| *LaABCG38* | 2.51 | 0.05 | 6.45 | 2.67 | 0.12 | 1.96 | 4.1 |
| *LaABCG39* | 0.35 | 0.69 | 0.35 | 0.17 | 0.69 | 0.21 | 0.46 |
| *LaABCG40* | 0.26 | 1.07 | 0.26 | 1.04 | 1.54 | 0.26 | 0.95 |
| *LaABCG41* | 0.1 | 0.02 | 0.1 | 0.02 | 0.1 | 0.1 | 0.1 |
| *LaABCG42* | 0.03 | 0.1 | 0.08 | 0.11 | 0.1 | 0.15 | 0.06 |
| *LaABCG43* | 0.02 | 0.39 | 0.1 | 2.51 | 0.68 | 0.09 | 4.29 |
| *LaABCG44* | 0.24 | 0.07 | 0.02 | 0.36 | 0.18 | 0.25 | 0.58 |
| *LaABCG45* | 0.1 | 0.1 | 0.1 | 0.1 | 0.1 | 0.1 | 0.1 |
| *LaABCG46* | 0.97 | 0.56 | 0.68 | 0.41 | 1.96 | 0.77 | 0.3 |
| *LaABCG47* | 0.97 | 0.56 | 0.68 | 0.41 | 1.96 | 0.77 | 0.3 |
| *LaABCG48* | 0.08 | 0.21 | 0.45 | 0.23 | 0.58 | 0.26 | 0.29 |
| *LaABCG49* | 3.86 | 0.16 | 1.42 | 0.26 | 0.12 | 5.36 | 0.41 |
| *LaABCG50* | 0.1 | 0.1 | 0.1 | 0.1 | 0.1 | 0.1 | 0.1 |
| *LaABCG51* | 17.55 | 1.86 | 13.09 | 6.82 | 4.2 | 12.21 | 6.06 |
| *LaABCG52* | 5.9 | 0.09 | 0.49 | 0.53 | 0.06 | 2.39 | 0.39 |
| *LaABCG53* | 15.74 | 3.97 | 27.61 | 17.09 | 4.42 | 20.55 | 20.16 |
| *LaABCG54* | 140.41 | 126.37 | 47.31 | 42.69 | 47.43 | 77.38 | 33.39 |
| *LaABCG55* | 0.02 | 14.34 | 0.1 | 2.99 | 25.07 | 0.02 | 2.97 |
| *LaABCG56* | 5.33 | 0.01 | 2.75 | 0.38 | 0.01 | 1.02 | 0.19 |
| *LaABCG57* | 205.1 | 144.22 | 85.64 | 164.66 | 79.15 | 168.52 | 193.31 |
| *LaABCG58* | 90.1 | 11.35 | 28.1 | 8.53 | 7.11 | 55.3 | 7.22 |
| *LaABCG59* | 0.4 | 6.22 | 0.46 | 2.72 | 7.72 | 0.47 | 2.33 |
| *LaABCG60* | 4.39 | 0.66 | 2.53 | 12.03 | 7.01 | 4.04 | 16.99 |
| *LaABCG61* | 110.2 | 8.55 | 76.88 | 83.62 | 16.02 | 94.4 | 86.9 |
| *LaABCG62* | 0.48 | 0.02 | 0.32 | 0.07 | 0.28 | 0.75 | 0.29 |
| *LaABCG63* | 0.07 | 0.1 | 0.17 | 0.02 | 0.1 | 0.28 | 0.05 |
| *LaABCG64* | 0.02 | 0.02 | 0.03 | 0.1 | 0.01 | 0.01 | 0.01 |
| *LaABCG65* | 0.01 | 0.1 | 0.02 | 0.12 | 0.1 | 0.08 | 0.28 |
| *LaABCG66* | 5.9 | 0.1 | 0.26 | 0.22 | 0.11 | 2.74 | 0.3 |
| **La; Lalb** |  |  |  |  |  |  |  |
| **LP; Low phosphorus, CK; Phosphorus sufficient, CR; Cluster root, L; leaf, RT; Root tip, R; Root, S; Stem** | | | | | | | |
