# Supplementary material for: Identification of ABC transporter G subfamily in white lupin and functional characterization of L.albABGC29 in phosphorus use
Source: BMC Genomics. 2021 Oct 6;22:723. doi: 10.1186/s12864-021-08015-0 (PMC8495970; doi:10.1186/s12864-021-08015-0)
Supplement: Supplementary file 11 — Additional file 11:. Expression pattern of ABCG subfamily members among different plant tissues under control (CK: phosphorus sufficient) and low phosphorus (LP) conditions. Fragment per kilobase of exon model per million mapped read (FPKM) values were used to visualize the gene expression on the heat map using R statistical online tool using RColorBrewer package. All the values are log2 transformed. LP-L/CK-L; leaf, CK-S/LP-S; stem, LP-RT; root tip, LP-CR; cluster root, and CK-R; root. [file 12864_2021_8015_MOESM11_ESM.docx]

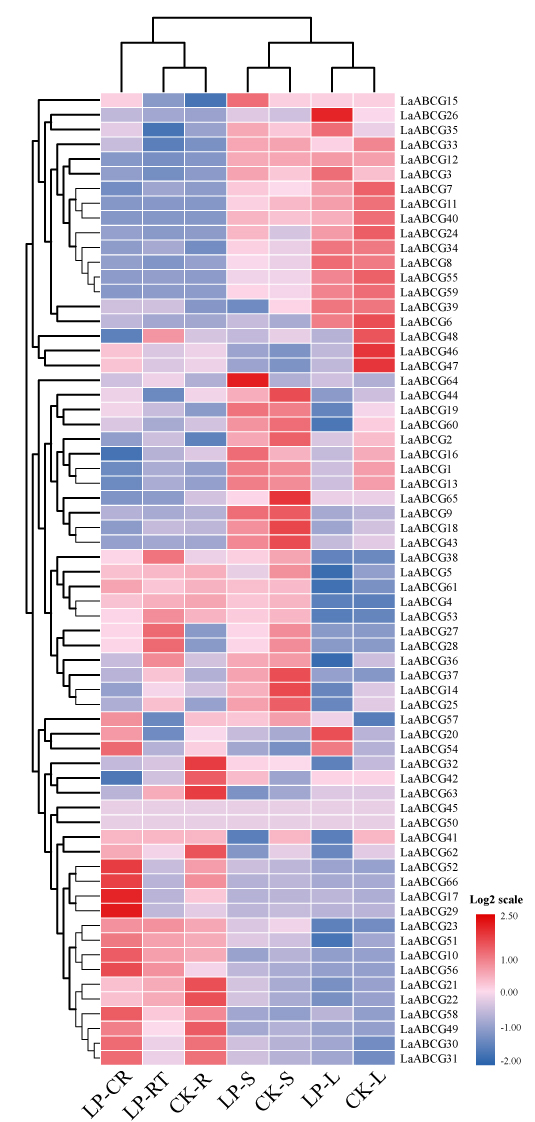
 **Additional file 11.** Expression pattern of ABCG subfamily members among different plant tissues under control (CK: phosphorus sufficient) and low phosphorus (LP) conditions. Fragment per kilobase of exon model per million mapped read (FPKM) values were used to visualize the gene expression on the heat map using R statistical online tool using RColorBrewer package. All the values are log2 transformed. LP-L/CK-L; leaf, CK-S/LP-S; stem, LP-RT; root tip, LP-CR; cluster root, and CK-R; root.
