# Supplementary material for: Identification of ABC transporter G subfamily in white lupin and functional characterization of L.albABGC29 in phosphorus use
Source: BMC Genomics. 2021 Oct 6;22:723. doi: 10.1186/s12864-021-08015-0 (PMC8495970; doi:10.1186/s12864-021-08015-0)
Supplement: Supplementary file 12 — Additional file 12:. Primers used for qPCR [file 12864_2021_8015_MOESM12_ESM.doc]

| **Additional file 12 Primers used for qPCR** | | | |  |
| --- | --- | --- | --- | --- |
| **Gene name** | **Target Plant** | **Forward primer (5’- 3’)** | **Reverse primer** **(5’- 3’)** | **Product size (bp)** |
| *Ubiquitin* | *L. albus* | GTCCACACTCCACCTTGTGC | GAGGAATGCCCTCCTTGTCCT | 149 |
| *Actin* | *O. sativa* | CCAGCAGATGTGGATTGCCA | CGGCGATAACAGCTCCTCTT | 119 |
| *LaBCG29* | *L. albus* and  *O. sativa* | TACTCCCCCTGGGCCTATTC | CGCGGACCAATAGTAACCCA | 117 |
